# Chromosomal instability shapes the tumor microenvironment of esophageal adenocarcinoma via a cGAS–chemokine–myeloid axis

**DOI:** 10.1126/sciadv.aeb1611

**Published:** 2026-03-11

**Authors:** Bruno Beernaert, Rose L. Jady-Clark, Parin Shah, Erik Ramon-Gil, Nora M. Lawson, Zack D. Brodtman, Somnath Tagore, Frederik Stihler, Alfie S. Carter, Shannique Clarke, Tong Liu, Winston M. Zhu, Juliet E. Martin, Erkin Erdal, Alistair Easton, Leticia Campo, Molly Browne, Stephen Ash, Rabial Q. Raja, Nicola Waddell, Tom Crosby, Simon R. Lord, Derek A. Mann, Ignacio Melero, Carlos E. de Andrea, Andréa E. Tijhuis, Floris Foijer, Ester M. Hammond, Kadir C. Akdemir, Jack Leslie, Benjamin Izar, Eileen E. Parkes

**Affiliations:** ^1^ Department of Oncology, University of Oxford, Oxford, UK.; ^2^ Centre for Immuno-Oncology, Nuffield Department of Medicine, University of Oxford, Oxford, UK.; ^3^ Department of Medicine, Division of Hematology and Medical Oncology, Columbia University Irving Medical Center, Herbert Irving Comprehensive Cancer Center, New York, NY, USA.; ^4^ Faculty of Medical Sciences, Newcastle University, Newcastle, UK.; ^5^ Department of Neurosurgery, Division of Surgery, The University of Texas MD Anderson Cancer Center, Houston, TX, USA.; ^6^ Translational Histopathology Laboratory, University of Oxford, Oxford, UK.; ^7^ Ludwig Institute for Cancer Research, University of Oxford, Oxford, UK.; ^8^ QIMR Berghofer Medical Research Institute, Brisbane, Australia.; ^9^ Velindre Cancer Centre, Velindre Hospital, Cardiff, UK.; ^10^ Departments of Pathology, Oncology and Immunology, Clínica Universidad de Navarra (CCUN), Pamplona, Spain.; ^11^ CIBERONC, Madrid, Spain.; ^12^ European Research Institute for the Biology of Ageing, University of Groningen, Groningen, Netherlands.

## Abstract

Chromosomal instability (CIN), a pervasive feature of esophageal adenocarcinoma (EAC), drives tumor aggressiveness and metastasis. CIN stimulates the cGAS–STING pathway, typically linked to antitumor immunity. However, despite the high CIN burden in EAC, the cGAS–STING pathway remains largely intact. To address this paradox, we interrogated multiple esophageal cancer models, finding myeloid-attracting chemokines—with *CXCL8* as a prominent hit—as conserved CIN-driven targets in EAC. Using multiplexed immunofluorescence microscopy, we quantified ongoing CIN in human EAC tumors by measuring cGAS-positive micronuclei, validated by whole-genome sequencing. Coupling in situ CIN detection with single-nucleus RNA sequencing and multiplex immunophenotyping of human EAC, we link CIN to tumor-intrinsic innate immune activation, *CXCL8* expression, and myeloid cell–mediated immunosuppression. In patients with EAC, CIN^high^, myeloid-dominated tumors correlate with poor outcomes and aberrant cGAS–STING signaling. These insights explain the counterintuitive maintenance of cGAS–STING and highlight the disruption of the CIN–cGAS–inflammation axis as a potential therapeutic strategy in EAC.

## Teaser

Chromosomal chaos rewires immune defense into tumor-promoting inflammation.

## INTRODUCTION

Esophageal adenocarcinoma (EAC) is an aggressive and frequently lethal cancer, with 5-year survival rates of less than 20% (*1*). Chromosomal instability (CIN) is a defining characteristic of EAC, with 97% of cases reported as belonging to the CIN subtype in the largest study to date of genomic characterization of gastroesophageal cancers (*2*). Manifestations of CIN, including chromosomal alterations, aneuploidies, and extrachromosomal DNA, occur early in the development of EAC, arising at nonmalignant stages of the precursor lesion Barrett’s esophagus (BE), and are associated with increased risk of progression to carcinoma (*3*–*6*). Genomic dysregulation from the earliest stages of development of EAC results in an almost ubiquitous mutational inactivation of *TP53* commonly associated with the loss of *CDKN2A* (*7*, *8*). Loss of these cell-cycle checkpoints permits the accumulation of oncogenic copy number alterations and genomic catastrophes, such as whole-genome doubling and chromothripsis (*9*, *10*), resulting in the chromosomal aberrations and complex genomic rearrangements observed in established EAC.

The chronic inflammatory background on which BE develops likely influences the pattern of immune infiltration subsequently observed in the tumor microenvironment (TME) of EAC. Certain chemokines and cytokines, including ligands to the myeloid receptors C-X-C motif chemokine receptors 1 and 2 [CXCR1/2; CXCL1, CXCL2, CXCL4, CXCL6, and CXCL8/interleukin-8 (IL-8)], IL-6 and IL-1β, have been reported to demonstrate a stepwise increase in their expression paralleled with disease progression (*11*, *12*). In addition, microenvironmental shifts with declining CD8^+^ T cells and the enrichment of myeloid and T regulatory cells are associated with progression from BE to EAC (*13*, *14*).

In CIN settings, we and others have reported the chronic stimulation of the innate immune cyclic GMP (guanosine 5′-monophosphate)-AMP (adenosine 5′-monophosphate) synthase (cGAS)-stimulator of interferon (IFN) genes (STING) pathway (*15*–*18*). Chromosome segregation errors frequently lead to the formation of cytoplasmic structures known as micronuclei (MN) containing whole chromosomes or chromosome fragments that lag behind during anaphase. These mis-segregated chromosomes recruit their own structurally unstable nuclear envelope, which frequently ruptures, with resultant exposure of nucleic acids to the cytosol (*19*). The double-stranded DNA (dsDNA) sensor cGAS detects cytoplasmic DNA, producing the second messenger 2′3′-cGAMP for subsequent activation of STING in an autocrine and paracrine manner. However, whether DNA within MN consistently activates cGAS remains an area of active debate, with recent studies suggesting that the link between MN and cGAS–STING activation may be correlative rather than causal (*20*–*23*).

The subsequent chemokine cascade, when acutely activated, results in antitumor immune responses (*24*). However, in the chronic setting, a paradoxical protumorigenic impact can be observed, with increased IL-6 secretion promoting tumor cell survival (*25*) and up-regulation of the cGAMP hydrolase ectonucleotide pyrophosphatase/phosphodiesterase 1 (ENPP1), which results in augmented extracellular adenosine and causes immunosuppression (*16*). Furthermore, chronic stimulation results in rewiring of the STING pathway, fostering survival and metastasis via noncanonical nuclear factor κB (NF-κB) signaling and endoplasmic reticulum (ER) stress responses (*15*, *17*).

However, the impact of CIN on the immune landscape in EAC is unknown. We hypothesized that the interaction between cancer and stromal cells, mediated by chronic cGAMP production or cGAS–STING–driven chemokine signaling, may profoundly influence the TME in CIN^high^ cancers and promote tumor aggressiveness while blunting therapeutic efficacy. Here, we provide evidence for CIN causing chronic cGAS–STING activation that enhances the expression of chemokines attracting protumor inflammatory infiltrates. Therefore, understanding the consequences of CIN-driven cGAS stimulation provides insights into mechanisms whereby this axis could be disrupted for patient benefit.

## RESULTS

### EAC maintains cGAS–STING despite high burdens of CIN

**Fig. 1.**
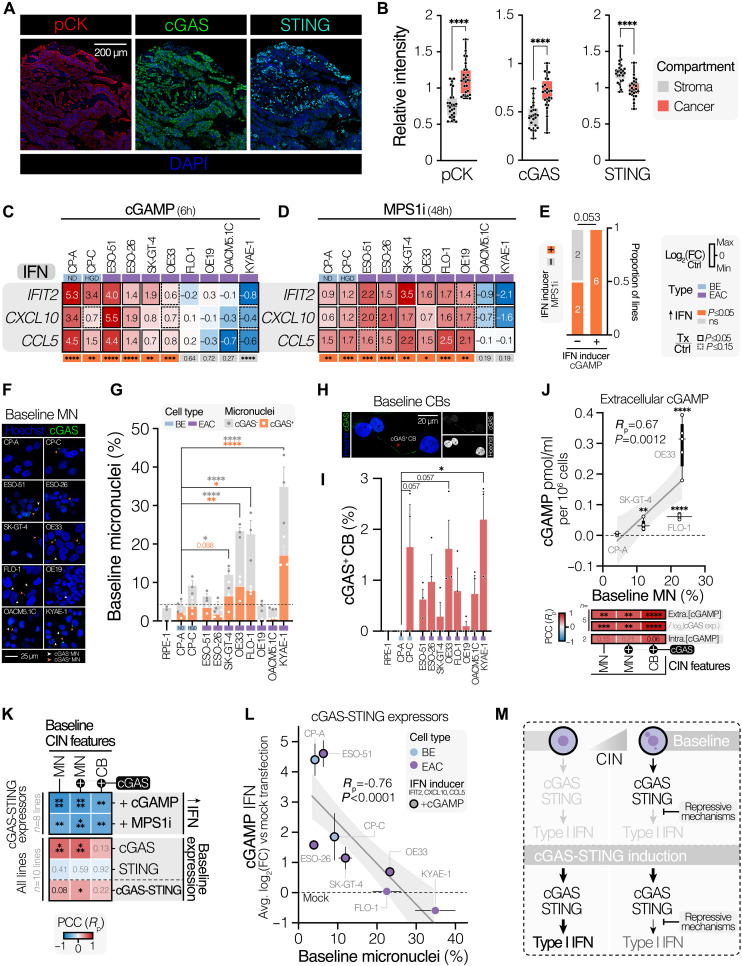
cGAS–STING functionality and CIN are prevalent features of EAC. (**A**) High-resolution image of a human EAC biopsy stained for cGAS, pCK, STING, and DAPI (DNA). Scale bar, 200 μM. (**B**) Quantification of (A). Average cancer versus stromal compartment marker intensities across EAC biopsies. Boxes: Median ± IQR (interquartile range). Whiskers: Minimum-maximum. Wilcoxon matched-pairs signed-rank test. (**C** and **D**) Heatmap of changes in mRNA abundance for “IFN” genes after (C) 2′3′-cGAMP (2 μg/ml) transfection or (D) MPS1i (0.5 μM) treatment, measured by RT-qPCR (*n* = 3). Two-sided paired *t* test. “IFN inducers”: Lines exhibiting significant overall IFN gene up-regulation. h, hours. (**E**) Relationship between IFN induction following 2′3′-cGAMP or MPS1i treatment in esophageal lines. Significant IFN inducers (+); noninducers (−). Two-sided chi-square test. (**F**) Confocal images of baseline esophageal lines, showing cGAS^−^/cGAS^+^ MN. Scale bars, 25 μm. (**G**) Quantification of baseline cGAS^−^/cGAS^+^ MN in esophageal lines. Bars: Means ± SEM (*n* = 3), ~≥100 cells per experiment. ANOVA, FDR-based correction. (**H**) Confocal image of baseline SK-GT-4 cells. Scale bar, 20 μm. CBs, chromatin bridges. (**I**) Quantification of baseline cGAS^+^ bridges in esophageal lines. Bars: Means ± SEM (*n* = 3, ~≥100 cells per experiment). ANOVA, FDR correction. (**J**) Upper: Correlation between extracellular 2′3′-cGAMP and MN frequency across select lines. Boxes represent the median ± IQR (*n* = 5). Horizontal error bars: ±SEM (*n* = 3). The regression line, 95% CI (confidence interval), *R*_p_, and *P* value are shown. Significance (CP-A versus EAC lines): Unpaired two-tailed *t* test. Lower: Correlations (Pearson) between CIN features and extracellular/intracellular 2′3′-cGAMP, including *cGAS* expression–normalized levels. (**K**) Correlations (Pearson) between CIN features and *cGAS/STING* expression (RT-qPCR) and treatment (MPS1i or 2′3′-cGAMP)–induced IFN induction. (**L**) Correlation (Pearson) between baseline MN and IFN gene responses to 2′3′-cGAMP in cGAS–STING expressors (fig. S1E). Regression line, 95% CI. Error bars: Means ± SEM (*n* = 3). (**M**) Model summarizing the relationship between CIN and cGAS–STING responsiveness. ****P ≤ 0.0001, ***P ≤ 0.001, **P ≤ 0.01, and *P ≤ 0.05.

Given the postulated central role of cGAS–STING in promoting antitumor immune responses, some cancers have been reported to suppress cGAS–STING via epigenetic silencing (*26*, *27*). However, analysis of genomic, methylation, and transcriptomic data from primary esophageal cancers from The Cancer Genome Atlas (TCGA) demonstrated a maintenance of *cGAS* and *STING* expression across both esophageal squamous cell carcinoma and EAC tumors versus matched normal tissue (fig. S1, A to C). Broad expression maintenance of *cGAS* and *STING* in esophageal lines was reflected in vitro, both within the Cancer Cell Line Encyclopedia (CCLE; fig. S1D) and across a panel of 10 esophageal cell lines (fig. S1E), comprising cell lines derived from nondysplastic and high-grade dysplastic BE tissue (CP-A and CP-C, respectively), as well as EAC. Multiplex immunofluorescence (mIF)–based profiling of 24 treatment-naïve EAC biopsy samples confirmed cGAS and STING protein expression in both malignant and stromal cell compartments in EAC, with cGAS expression predominant in the malignant compartment and STING in the stroma (Fig. 1, A and B).

To assess whether the maintenance of cGAS–STING expression in BE and EAC lines corresponded to functional type I IFN signaling upon STING pathway induction, we treated cells with the STING agonist 2′3′-cGAMP or induced CIN using the monopolar spindle kinase 1 (MPS1) inhibitor (MPS1i) reversine (fig. S2, A and B) and monitored the expression of the IFN-stimulated genes (ISGs) *CCL5*, *CXCL10*, and *IFIT2*—key targets of STING-driven type I IFN signaling (Fig. 1, C and D). Nontransformed, genomically stable BE lines (CP-A and CP-C) exhibited strong induction of IFN-responsive genes (referred to as “IFN” genes) in response to STING-activating treatments, whereas EAC lines exhibited more variable responses, including an expected absence of 2′3′-cGAMP–driven IFN gene induction in cell lines with suppression of core cGAS–STING machinery expression (OE19 and OACM5.1C; fig. S1E). Some EAC lines also demonstrated failure of IFN gene induction in response to 2′3′-cGAMP in lines with competent cGAS–STING expression levels (FLO-1 and KYAE-1). Notably, some cell lines that did not demonstrate IFN gene expression in response to direct STING agonism through 2′3′-cGAMP treatment maintained competent IFN signaling in response to MPS1 inhibition (e.g., OE19 and FLO-1), suggestive of STING-independent modes of CIN-driven IFN gene activation. Nevertheless, IFN induction capacity in response to cGAMP exposure coincided with IFN up-regulation following MPS1 inhibition, suggesting a relationship between STING activation–driven and CIN-driven IFN induction capacities (Fig. 1E).

Constitutive CIN has recently been proposed to drive the adaptive rewiring of signaling downstream of STING, enabling tumor cells to evade the deleterious effects of type I IFN activation (*17*). To assess the prevalence and extent of CIN in EAC cells and evaluate its relationship with IFN gene induction capacity, we next measured the frequency of proposed cGAS-activating CIN features, including MN, cGAS^+^ MN (*28*, *29*), and cGAS^+^ chromatin bridges (CBs) (*30*), under baseline conditions by immunofluorescence (Fig. 1, F to I).

cGAS^+^ MN and chromatin bridges were observed to be similarly low in both the nondysplastic BE CP-A line and the genomically stable, noncancerous human telomerase reverse transcriptase (hTERT) RPE-1 cell line. However, CIN feature frequencies differed significantly across high-grade dysplastic BE as well as EAC lines, with four of eight EAC lines (OE33, SK-GT-4, FLO-1, and KYAE-1) exhibiting significantly elevated MN or chromatin bridge burdens over the CP-A cell line. Baseline incidences of CIN features were found to be significantly intercorrelated (fig. S2C). CIN features also correlated with tonic cGAS activity, as inferred through 2′3′-cGAMP enzyme-linked immunosorbent assay (ELISA), irrespective of *cGAS* expression, across profiled cell lines (Fig. 1J). In contrast, baseline CIN feature burdens were strongly anticorrelated with IFN gene induction capacity following either 2′3′-cGAMP or MPS1i stimulation, indicating diminished IFN pathway engagement in response to stimulation of cGAS–STING in CIN^high^ cells (Fig. 1, K and L).

Together, these results suggest that EAC cells broadly maintain cGAS and STING expression and retain at least some degree of cGAS–STING–IFN pathway functionality. However, the magnitude of effective IFN pathway response upon cGAS–STING stimulation is inversely proportional to the inherent CIN burden to which tumor cells are subjected in vitro (Fig. 1M).

### CIN-driven cGAS–STING controls a distinct subset of immune target genes in EAC

To interrogate the nature of CIN-induced cGAS–STING innate immune responses in EAC cells, we abrogated cGAS using CRISPR-Cas9–mediated knockout (cGAS^KO^) in a subset of EAC lines, selected specifically for their high inherent micronucleation rates and differential type I IFN induction capacities (OE33, FLO-1, and SK-GT-4). cGAS^KO^ clones showed a loss of cGAS at the protein level as well as a significantly diminished cGAS mRNA abundance (fig. S3, A to C), and failed to produce 2′3′-cGAMP in response to both CIN induction through MPS1 inhibition and direct cGAS agonism through G_3_-YSD [a Y-form DNA-selective cGAS agonist (*31*)] transfection compared to respective empty-vector (Cas9) clones (fig. S3D).

**Fig. 2.**
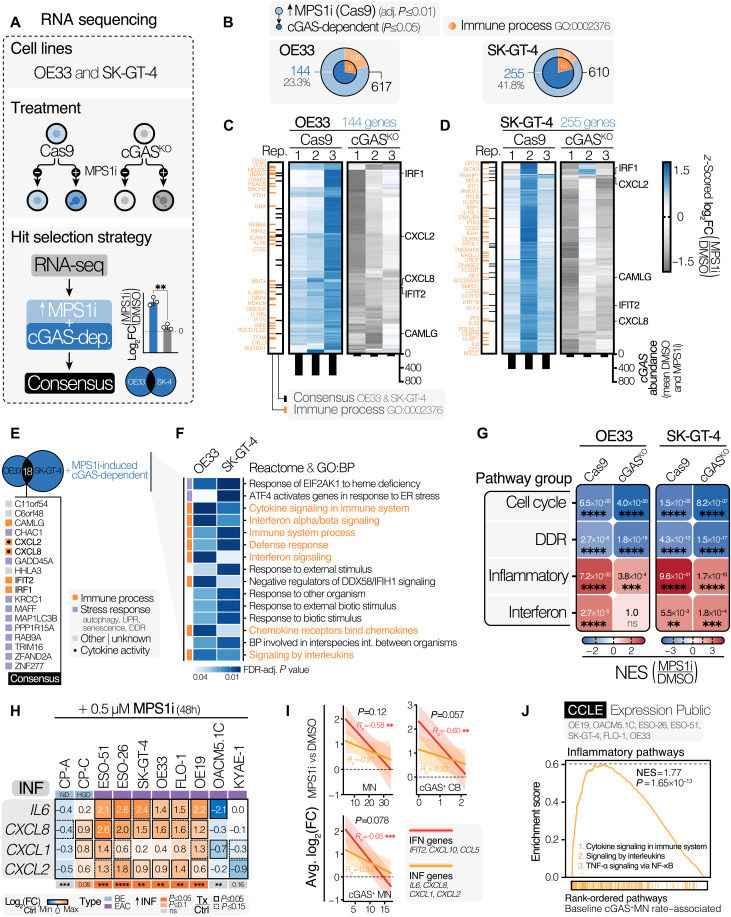
CIN-driven cGAS–STING activation drives inflammatory gene expression in EAC cells. (**A**) RNA-seq strategy to identify cGAS-dependent CIN-induced genes. (**B**) Venn diagram of CIN/MPS1i–up-regulated hits (outer circle) and cGAS-dependent subset (inner circle). “Immune System Process” hits highlighted. (**C** and **D**) Heatmaps of log_2_ FCs (MPS1i versus DMSO) of cGAS-dependent CIN-driven genes for (C) OE33 and (D) SK-GT-4 Cas9 and cGAS^KO^ cells, *n* = 3 biological repeats. Consensus and “Immune System Process” genes are highlighted. Average cGAS expression across (DMSO + MPS1i) replicates is plotted. Color maps to *z*-scored log_2_(FC). (**E**) Overlap between CIN-induced cGAS-dependent genes in OE33 and SK-GT-4 cells. Consensus hits are annotated with biological functions. (**F**) Heatmap of overrepresentation analyses of MPS1i-induced cGAS-dependent genes in OE33 and SK-GT-4 cells. The top 15 pathways are shown. Orange pathways have immune-promoting functions. (**G**) Heatmap of GSEA outputs, showing MPS1i-induced pathway changes in Cas9 and cGAS^KO^ clones. Focus on pathways relating to the cell cycle, DDR, INF, or IFN. Color maps to the normalized enrichment score (NES). FDR *q* values (FDRq) are shown. (**H**) Heatmap of MPS1i-induced inflammatory target mRNA abundance changes in esophageal cell lines. Expression determined by RT-qPCR. Color maps to average log_2_(FC), *n* = 3 independent experiments. Data analyzed by two-sided paired *t* test. Significance of overall INF gene induction capacity determined via two-sided paired *t* test, comparing *Z*-normalized expression values of treated versus control samples. (**I**) Scatterplots showing the relationship between baseline CIN features and immune target induction upon MPS1i across cGAS–STING–expressing cell lines. The simple linear regression line, 95% confidence intervals, *R*_p_ values, Pearson *P* values, and linear model interaction term *P* values are shown. (**J**) GSEA enrichment plot showing the enrichment of inflammatory pathway gene expression with increasing baseline cGAS^+^ MN rates across indicated EAC cell lines. ****P ≤ 0.0001, ***P ≤ 0.001, **P ≤ 0.01, and *P ≤ 0.05; ns, not significant.

To identify cGAS-dependent CIN-induced targets, we profiled the transcriptomes of Cas9 control and cGAS^KO^ clones of OE33 and SK-GT-4 cells with or without MPS1i treatment (Fig. 2A). A substantial proportion of CIN-up-regulated genes in both cell lines was found to be dependent on cGAS function, with 144 (23.3%) and 255 (41.8%) genes in OE33 and SK-GT-4 cells, respectively, exhibiting a significantly diminished up-regulation in cGAS^KO^ backgrounds (Fig. 2, B to D, and fig. S3, E to H). Of these, approximately one in five had known immune or inflammatory functions. Eighteen consensus hits were identified, including the CXCR1/2–acting chemokines *CXCL8* (encoding IL-8) and *CXCL2* (also known as MIP2-α), the IFN pathway genes *IRF1* and *IFIT2*, and components of the unfolded protein response ER stress and autophagy pathways [e.g., *CHAC1*, *ZFAND2A*, *TRIM16*, and *MAP1LC3B* (known as LC3)], potentially reflecting reported noncanonical roles for cGAS in these pathways (Fig. 2E) (*32*, *33*). Overrepresentation analysis indicated that CIN-induced cGAS-dependent genes in both OE33 and SK-GT-4 were enriched for immune-related functions, including IFN and chemokine/cytokine signaling pathways (Fig. 2F). Gene set enrichment analysis (GSEA) of MPS1i-induced transcriptional changes revealed that the engagement of inflammatory response pathways (including NF-κB, chemokine, cytokine, and inflammatory signaling gene sets), rather than IFN pathways [including IFN, ISG, and JAK (Janus kinase)-STAT (signal transducer and activator of transcription) gene sets] was most pronounced upon CIN induction and most markedly diminished by cGAS deletion in both cell lines (Fig. 2G).

Several cGAS-dependent immune hits (*IL6*, *CXCL8*, *CXCL2*, and *IFIT2*) were confirmed using reverse transcription quantitative polymerase chain reaction (RT-qPCR), demonstrating cGAS-dependent CIN-induced up-regulation across multiple independent clones (fig. S3I). Similarly, MPS1i-induced secretion of IL-8 was measured by ELISA in cell supernatants and confirmed to be significantly reduced in cGAS^KO^ cell lines (fig. S3J).

CIN-induced expression up-regulation of inflammation-associated cytokines (*IL6*, *CXCL8*, *CXCL1*, and *CXCL2;* “INF” genes) appeared broadly conserved across BE and EAC lines (in 7 of 10 lines; Fig. 2H) and did not exhibit the same degree of baseline CIN-associated suppression seen with IFN-responsive genes (Fig. 2I). Baseline inflammatory pathway activity [inferred from CCLE RNA sequencing (RNA-seq) data] was commensurate with the inherent cGAS^+^ MN burdens of EAC lines, as determined via immunofluorescence (IF)–based profiling (Fig. 2J), suggestive of tonic CIN-driven inflammatory activity in cell lines with high inherent micronucleation rates. Nevertheless, the magnitude of inflammatory gene up-regulation upon CIN induction observed across cell lines was modest (maximum sixfold; Fig. 2H), suggesting that low-level “smoldering” inflammation in CIN cells may result in an impaired ability to induce further inflammation in response to further transient CIN increases.

In addition, CIN-induced inflammatory gene induction was still noted in the cGAS-nonexpressing line OE19 (fig. S1D) and in the STING pathway–impaired FLO-1 cell line (Fig. 1C) upon cGAS abrogation (fig. S3K), indicative of cGAS–STING–independent modes of CIN-driven inflammatory target induction. Collectively, our data support a model wherein CIN results in the constitutive expression of cGAS-dependent inflammatory mediators, although some inflammatory signaling is retained in certain CIN^high^ settings independent of cGAS status.

### CIN scales with inflammatory activity in an isogenic BE model

**Fig. 3.**
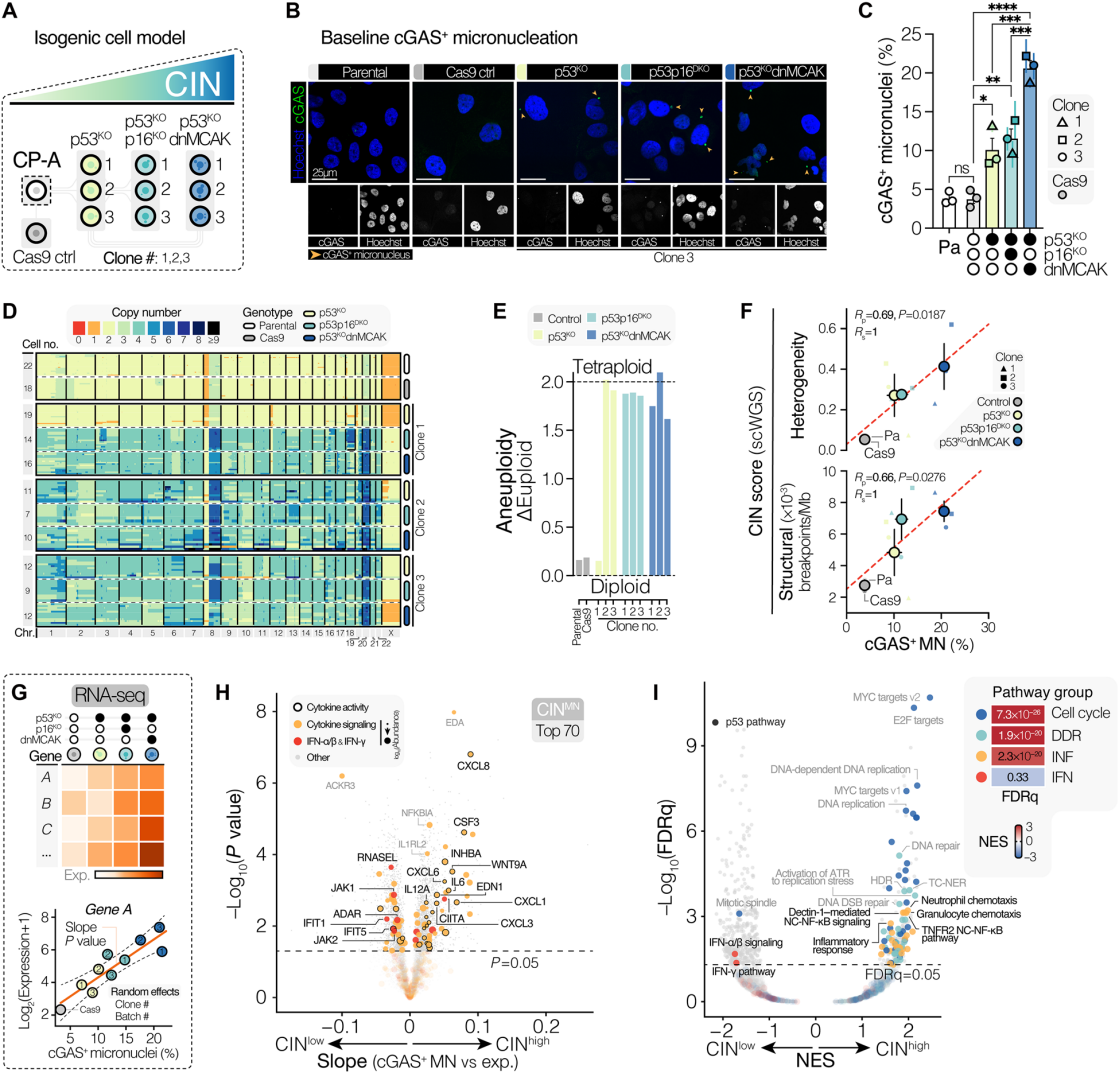
Inflammatory pathway activity scales with the inherent level of CIN in an isogenic BE cell line model. (**A**) Schematic representation of the isogenic CIN cell model, showing cell lines derived from the founder CP-A. (**B**) Confocal images showing baseline cGAS^+^ MN across lines. Exemplars are progenitor matched (clone 3). Scale bar, 25 μm. (**C**) Quantification of cGAS^+^ MN in parental CP-A cells and derived lines. Bars for p53^KO^, p53p16^DKO^, and p53^KO^dnMCAK genotypes: Means ± SEM (*n* = 3 clones), with each point showing the means ± SEM (*n* = 3, ≥100 cells per experiment). Shapes: Clone numbers for p53^KO^, p53p16^DKO^, and p53^KO^dnMCAK. ANOVA, FDR-based correction. (**D**) Heatmap of scWGS results. Each row represents a single cell. Sequenced cells per genotype displayed. Columns: Autosomes 1 to 22 and X chromosome. Color: Ploidy at the indicated locus. (**E**) Bar plots of inferred aneuploidy scores relative to euploid control. (**F**) Correlations between mean (genotype-level) cGAS^+^ MN versus CIN (scWGS-derived) metrics, including structural breakpoints per megabase (Mb) and between-cell karyotype heterogeneity. The regression line, *R*_p_ and Spearman coefficient (*R*_s_) values, and *P* values are shown. Data point shapes for p53^KO^, p53p16^DKO^, and p53^KO^dnMCAK: Clone number. (**G**) Analysis approach for identification of CIN-associated genes from RNA-seq data. (**H**) Volcano plot showing associations between gene expression and cGAS^+^ MN within the CP-A model. Genes involved in cytokine signaling (GO: 0019221), involved in IFN-α/β or IFN-γ signaling (R-HSA-909733 or R-HSA-877300), or with cytokine activity (GO: 0005125) are marked as indicated. For immune genes, dot size: Mean mRNA abundance. (**I**) Volcano plot of GSEA outputs showing pathways associated with cGAS^+^ MN burden in CP-A cells. Select pathways are categorized into cell cycle–, DDR-, INF-, or IFN response–related groups. The p53 pathway (MSigDB Hallmarks) is highlighted. The heatmap shows the extent of CIN enrichment of pathway groups. ****P ≤ 0.0001, ***P ≤ 0.001, **P ≤ 0.01, and *P ≤ 0.05; ns, not significant.

To ensure that our findings were a direct result of CIN and not due to the impact of drug-induced acute induction of CIN on cell viability, and to more closely model the chronicity of CIN in EAC, we developed isogenic esophageal cell line models with varying levels of steady-state CIN (fig. S4, A to E). Using the nondysplastic, hTERT-immortalized BE-derived CP-A cell line (*34*) as a founder model, we validated its reported *TP53* wild-type status and confirmed the possession of a functional cGAS–STING pathway (fig. S5, A to E). To mimic a genetic background permissive to high levels of CIN in this cell line, we first serially inactivated *TP53* and *CDKN2A* (p53^KO^ and p16^KO^, respectively) using CRISPR-Cas9–mediated gene editing (fig. S5F). Disruption of these pathways is among the most prevalent genetic events in EAC tumors and are typically early events in disease progression (*7*). Subsequently, we introduced a dominant-negative mutant form of the mitotic regulator MCAK (known as KIF2C; dnMCAK) on the p53^KO^ background to induce the high rate of chromosome mis-segregations encountered in EAC (Fig. 3A). Single-cell clones were selected using fluorescence-activated cell sorting, puromycin selection, and IF-based screening of baseline cGAS^+^ MN frequencies across clones (fig. S4, A to E). Selected clones were validated through amplicon sequencing of guide RNA target sites, challenge with Nutlin-3 and puromycin, and immunoblotting for p53 and MCAK (fig. S5, F to N).

To evaluate the extent of CIN in our isogenic cell line model, we quantified the baseline frequency of cGAS^+^ MN using IF. Deletion of *TP53* led to a significant increase in cGAS^+^ MN, which was further amplified by dnMCAK overexpression, with strong concordance between independent clones (Fig. 3, B and C). Additional deletion of *CDKN2A* (p53p16^DKO^) did not augment the observed CIN phenotype compared to p53^KO^ CP-A cells. Using shallow single-cell whole-genome sequencing (scWGS) (*35*), CP-A parental and Cas9 cells were confirmed to be largely karyotypically homogeneous and near-diploid (Fig. 3D). Whole-genome doubling was observed in two of three p53^KO^ clones and all successively derived clones (p53p16^DKO^ and p53^KO^dnMCAK clones; Fig. 3E). Elevated rates of CIN were reflected at the genomic level, with altered lines being increasingly characterized by genomic aberrations associated with chromosome mis-segregations, including an elevated frequency of inferred chromosomal breakpoints and increased karyotype heterogeneity across sequenced cells, scaling in a similar manner to observed cGAS^+^ MN rates (Fig. 3F).

We performed RNA-seq profiling of all cell lines of our CIN-isogenic model (Cas9, p53^KO^, p53p16^DKO^, and p53^KO^dnMCAK) to assess the impact of stably elevated CIN on transcriptomic profiles using a linear mixed-effects model to assess the relationship between gene expression and the observed cGAS^+^ MN frequency of each cell line (Fig. 3G). Similar to MPS1i treatment–induced changes, we again observed a significant scaling of expression of multiple inflammatory response-related cytokine and chemokine genes with baseline cGAS^+^ MN burden, including CXCR1/2 ligands (*CXCL8*, *CXCL1*, *CXCL3*, *CXCL6*, and *CXCL7*) as well as *IL6*, *CSF1*, and *CSF3* (Fig. 3H). Expression profiling of several CIN-associated chemokines (*CXCL8*, *IL6*, and *CXCL1*) through RT-qPCR confirmed a proportional increase with CIN burden, with a similar increase noted in IL-8 secretion as measured by ELISA in cell supernatants (fig. S6, A to C). At a pathway level, GSEA showed the broad enrichment of multiple inflammation-related pathways with increasing CIN, including neutrophil and granulocyte chemotaxis, cytokine-cytokine receptor interaction, and noncanonical NF-κB (Fig. 3I). Activation of noncanonical NF-κB signaling was also observed in CP-A p53^KO^dnMCAK cells, with an increased p52 protein abundance and p52/p100 ratio compared to parental CP-A cells (fig. S6, D and E).

To assess the degree to which in vitro transcriptional outcomes of CIN in CP-A cells reflected CIN as encountered in other cells and in EAC tumors, we selected the top 70 genes most strongly positively associated with cGAS^+^ MN burden (termed the CIN^MN^ signature; Fig. 3H and table S1) and evaluated the association between our in vitro model–derived CIN^MN^ signature scores and previously published orthogonal measures of CIN, including the transcriptional CIN70 signature score (referred to as CIN^70^) (*36*) and the whole-genome sequencing (WGS)–derived aneuploidy score (*37*). Using RNA-seq data from the CCLE, we identified a positive correlation between transcriptome-derived CIN^MN^ scores and baseline cGAS^+^ MN burdens across seven EAC cell lines (fig. S7A). In addition, across cell lines derived from multiple tumor sites in the CCLE, as well as in the EAC and the closely related chromosomally unstable stomach adenocarcinoma (STAD-CIN) TCGA cohorts, CIN^MN^ scores significantly positively associated with CIN^70^ and aneuploidy scores (fig. S7, B to D).

To assess whether observed correlations between CIN scores also reflected a similar underlying biology in CIN^high^ EAC tumors, we used GSEA to determine which pathways scaled with CIN^MN^, CIN^70^, and aneuploidy scores. All three CIN scores were associated with similar biological processes, including the expression of E2F targets, MYC targets, and G_2_-M checkpoints (fig. S7E). In keeping with our in vitro findings, CIN measures were consistently associated with increased expression of CXCR1/2 ligands. The inclusion of cGAS–STING expression scores as a covariate in our model enabled evaluation of genes that scaled with CIN in a manner dependent on cGAS–STING expression levels. Multiple chemokines and cytokines were found to correlate with CIN^MN^ in a cGAS–STING–dependent manner, including *CXCL8*, *IL6*, and *CSF3* (fig. S7F), accompanied by a broad enrichment in cytokine-related pathways, consistent with our in vitro modeling (fig. S7G). Together, these data are consistent with CIN as a driver of a distinct inflammatory response within the TME.

### MN burden in EAC tumors correlates with innate immune activity

To define the role of elevated CIN in tumor cell–intrinsic innate immune activation and microenvironmental inflammation, we performed single-nucleus transcriptomic analysis of human EAC tumors across the gradient of CIN. Here, we first refined a mIF-based approach to detect cGAS^+^ MN as a measure of ongoing CIN on the basis of our previous methodology (*16*). Formalin-fixed paraffin-embedded (FFPE) tumor sections were stained using a mIF panel consisting of antibodies (Abs) detecting cGAS and pan-cytokeratin (pCK) alongside the nuclear counterstain 4′,6-diamidino-2-phenylindole (DAPI), enabling the identification of cGAS^+^ MN in malignant EAC cells (fig. S8A). We quantified cGAS^+^ MN within the cancer cell compartment, normalizing to the tumor cell number per section using a semiautomated method that closely matched manual (“ground-truth”) counts (fig. S8B). Consistent with CIN as a hallmark of malignant cells, cGAS^+^ MN were largely confined to tumor cell compartments versus stromal areas (fig. S8C). In addition, cGAS^+^ MN scores showed high reproducibility across patient-matched biopsy and resection samples, with lower intrapatient variance compared to interpatient differences (fig. S8D).

Next, we conducted WGS on 12 matched tumor samples to quantify the extent of CIN inferred from genomic features and compare it to cGAS^+^ MN scores. We observed notable correlations between cGAS^+^ MN frequencies and WGS-derived measures of CIN, including the cumulative load of all structural variants (SVs), as well as specific SVs, including inversions, break-ends, duplications, and deletions (fig. S8, E and F).

**Fig. 4.**
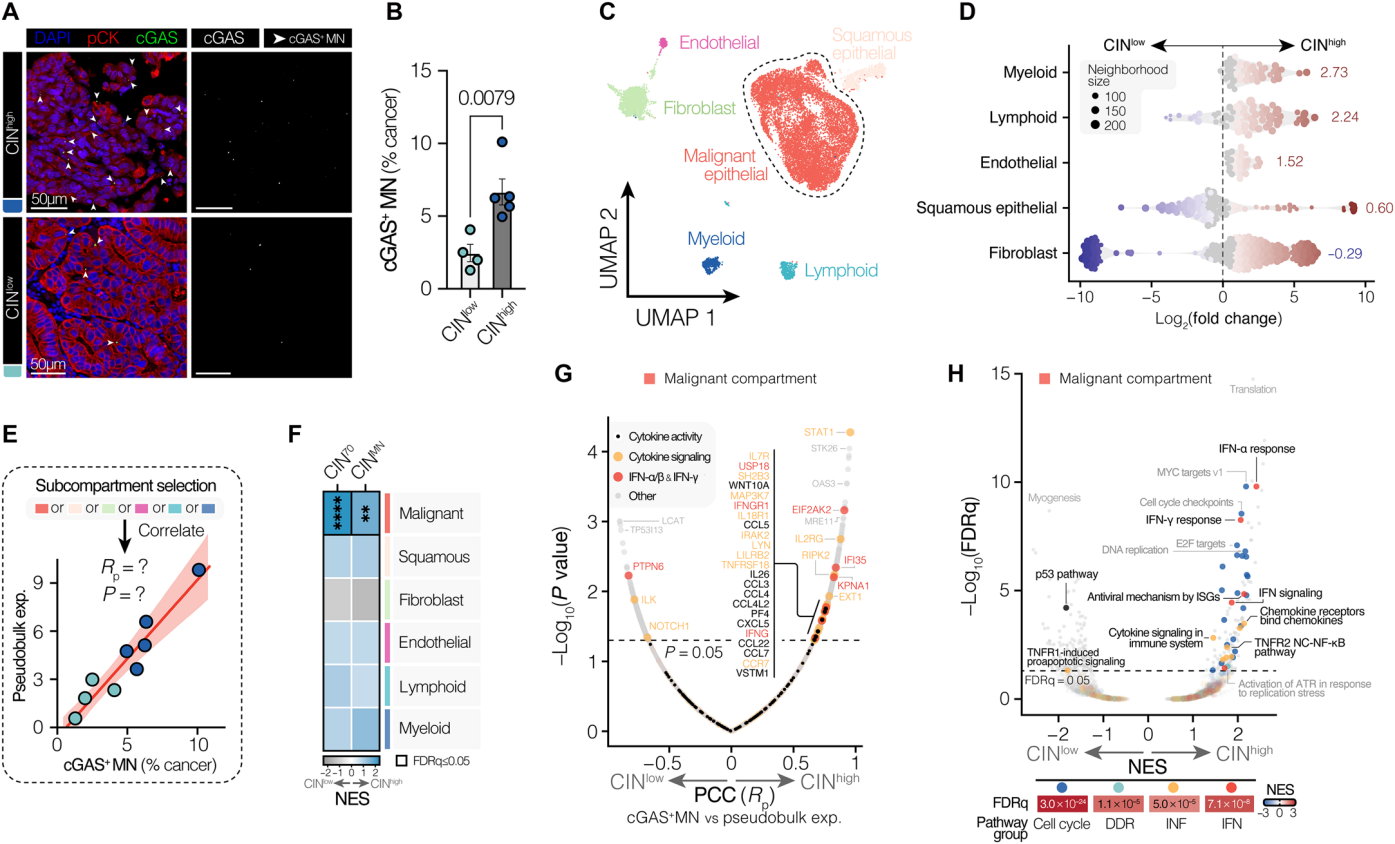
MN burden scales with innate immune activity in human EAC tumors. (**A**) Representative high-resolution images of human EAC tumors stained with DAPI (DNA) and indicated Abs, showing cGAS^+^ MN (white arrows) in CIN^high^ and CIN^low^ tumors shortlisted for snRNA-seq. Scale bar, 50 μM. (**B**) Tumoral cGAS^+^ MN frequencies in (CIN^high^/CIN^low^) tumors shortlisted for snRNA-seq. Bars represent the means ± SEM. Data were analyzed by a two-tailed unpaired *t* test. (**C**) UMAP dimensionality reduction of all cells remaining after quality control and filtering, colored by manually curated cell identity. (**D**) Beeswarm plot of Milo analysis outputs for nonmalignant cell clusters. The dot size corresponds to the neighborhood size. Directionality and weighted mean (shown) indicate the CIN category (CIN^high^/CIN^low^) in which there is a greater relative abundance of the indicated cell type. The *x* axis represents log_2_(FC) in relative cell abundance. (**E**) Hit selection strategy for identifying genes associated with tumoral cGAS^+^ MN frequencies in each cell compartment. (**F**) Heatmap of GSEA analysis querying the degree of CIN signature enrichment across cGAS^+^ MN–correlated genes in each cell compartment. Color maps to the normalized enrichment score. The cell border indicates an FDRq ≤ 0.05. (**G**) Volcano plot showing the association between gene expression and tumoral cGAS^+^ MN frequency in the malignant cell compartment. *R*_p_ values (*x* axis) and *P* values (*y* axis) are plotted for each gene. Genes involved in cytokine signaling (GO: 0019221), involved in IFN-α/β or IFN-γ signaling (R-HSA-909733 or R-HSA-877300), or with reported cytokine activity (GO: 0005125) are marked as indicated. (**H**) Volcano plot of GSEA outputs, showing the relationship between pathway enrichment and tumoral cGAS^+^ MN burden. Select pathways have been categorized into cell cycle–, DDR-, INF-, or IFN response–related pathways. The p53 pathway (MSigDB Hallmarks) has been highlighted. The heatmap shows the extent of CIN enrichment of broad pathway groups. ****P ≤ 0.0001 and **P ≤ 0.01.

Having validated our in situ CIN scoring approach, we shortlisted nine tumors spanning the CIN spectrum observed across 45 primary EAC tumors for single-nucleus RNA-seq (snRNA-seq; fig. S9, A and B). snRNA-seq was performed on FFPE sample–matched freshly frozen tumor samples using the 10x Genomics platform, sampling four CIN^low^ and five CIN^high^ tumors, with CIN^high^ tumors harboring, on average, 2.7 times the cGAS^+^ MN burden of CIN^low^ tumors (Fig. 4, A and B).

In total, we obtained the transcriptomes of 16,127 cells. Graph-based clustering of transcriptomes, followed by manual curation of subclusters, generated six major cellular compartments, comprising epithelial cells (malignant EAC cells and squamous epithelial keratinocytes), immune cells (lymphoid and myeloid cells), fibroblasts, and endothelial cells, which exhibited gene markers and pathway enrichment in line with assigned cluster identities (Fig. 4C and fig. S9, C to F). Malignant EAC cells (12,201 cells) comprised most detected cells. Both lymphoid and myeloid cells were enriched in tumors with higher preponderances of cGAS^+^ MN, suggesting that CIN^high^ tumors are more heavily immune infiltrated (Fig. 4D and fig. S9G). While abundant lymphocytic infiltrate is typically indicative of effective antitumor immune responses, our observation that CIN^high^ tumors are also strongly infiltrated with myeloid cells and tumor-associated macrophages suggests an elevated degree of myeloid-mediated immunosuppression.

To identify tumor cell–intrinsic expression patterns associated with tumoral CIN levels, we performed linear correlation analysis between microscopy-assessed cGAS^+^ MN frequencies and compartment-specific pseudobulked gene expression, focusing on the malignant cell compartment (Fig. 4E). We found that CIN^70^ and CIN^MN^ signatures were significantly and selectively enriched across CIN-correlated genes in the malignant compartment (Fig. 4F). This expected specificity to the malignant compartment supported the notion that this approach could identify cell type–specific CIN-associated expression patterns. Multiple cytokine and IFN signaling–related genes were strongly positively correlated with tumoral cGAS^+^ MN frequencies (Fig. 4G). These included *STAT1* as well as *IFNG* and ISGs including *OAS3* and *EIF2AK2* (encoding the double-stranded RNA sensor PKR). Immune targets that scaled with cGAS^+^ MN included *CCL3*, *CCL4*, *PF4*, the CXCR2 ligand *CXCL5*, and the CCR2 ligand *CCL7*. Up-regulation of immune targets was reflected at a pathway level, with significant enrichment of multiple immune pathways with increasing CIN, including IFN-α and IFN-γ response pathways as well as cytokine and noncanonical NF-κB signaling (Fig. 4H).

While a significant linear association between *CXCL8* and cGAS^+^ MN burden was not observed, the overall abundance of *CXCL8* mRNA and other in vitro cytokine hits (*CXCL1*, *CXCL2*, *CXCL3*, and *CSF3*) was broadly increased in the malignant compartments of CIN^high^ tumors (fig. S9H). Enhanced immune target expression in CIN^high^ tumors was further accompanied by the expression of immune checkpoints in the malignant compartment including *CD274* (PD-L1), *IDO1*, *CD47*, and the cGAS–STING checkpoint *ENPP1* (fig. S9H). Together with the increased expression of T cell exhaustion markers in the lymphoid compartment (fig. S9I), this is potentially indicative of ongoing evasion of both myeloid-mediated (e.g., CD47-SIRP1α phagocytosis checkpoint) and T cell–mediated tumor cell killing in CIN^high^ EAC.

### Mapping the TME of CIN^high^ tumors

**Fig. 5.**
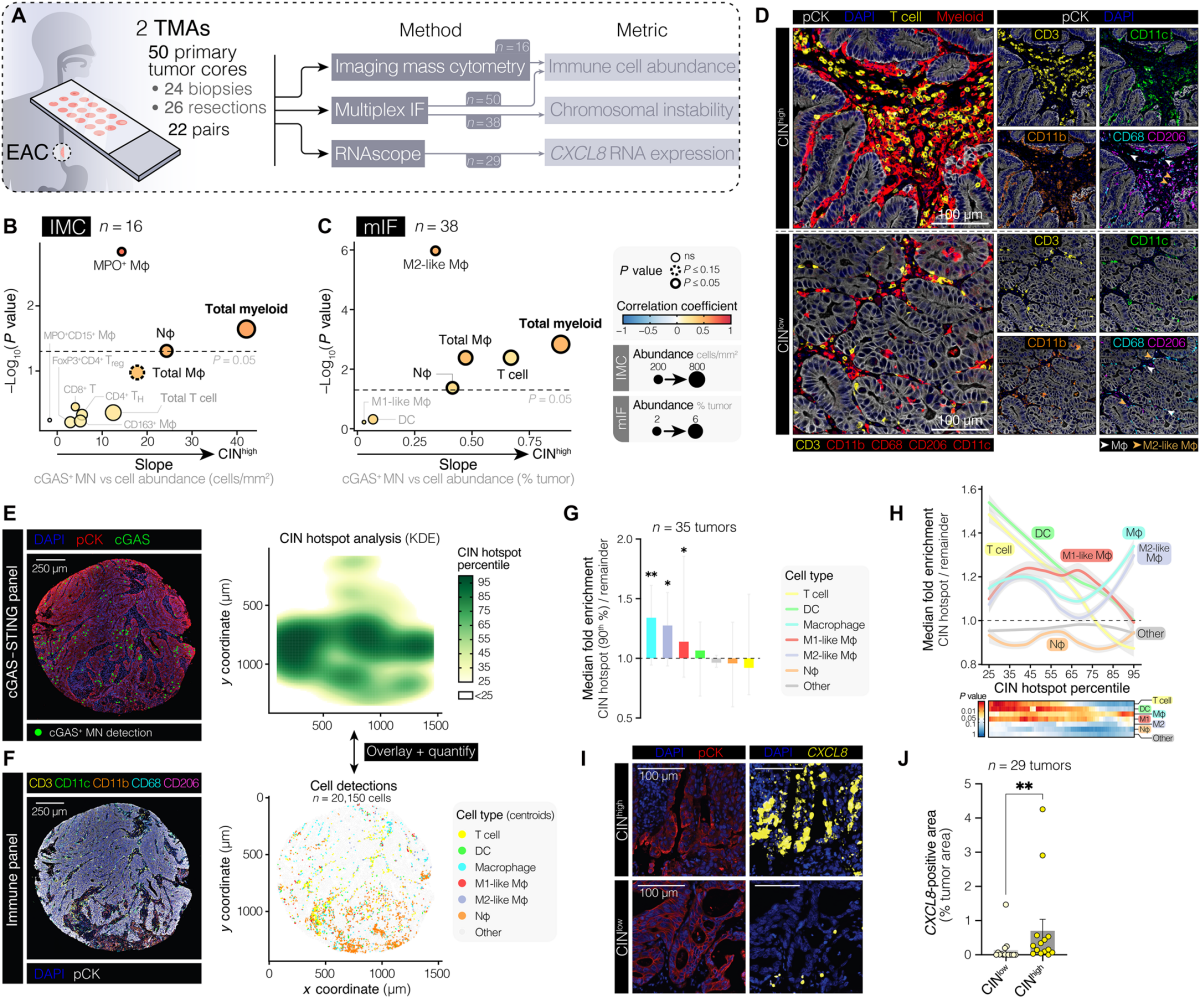
CIN is associated with intratumor myeloid cell inflammation in human EAC tumors. (**A**) EAC TME profiling workflow. (**B** and **C**) Volcano plots showing associations between immune cell subtypes and CIN (cGAS^+^ MN frequency) across (B) EAC biopsies queried by IMC and (C) pre- and posttreatment EAC tumors stained via mIF. The *y* axis indicates significance (Pearson correlation *P* value). The *x* axis denotes the slope of the association between CIN and cell abundance. The dot size represents the mean cell density/abundance across samples. The border indicates significance. Color maps to *R*_p_ for (B) and *R*_s_ for (C). (**D**) Representative high-resolution images of CIN^high^ and CIN^low^ EAC biopsies stained with Abs targeting CD3, pCK and myeloid markers, and DAPI (DNA). Macrophage examples are indicated with arrows. Scale bars, 100 μm. (**E**) Left panel: Representative image of a human EAC tumor stained with Abs targeting pCK and cGAS, as well as DAPI. The spatial positions of cGAS^+^ MN detections are highlighted. Scale bar, 250 μm. Right panel: Heatmap of quantized cGAS^+^ MN Kernel densities. (**F**) Left panel: Image of a tumor section adjacent to the section in (E), stained with an immune panel. Scale bar, 250 μm. Right panel: Spatial positions of classified cell centroids. (**G**) Fold enrichment in the relative immune cell abundance in cGAS^+^ MN–dense areas (“CIN hotspot”) versus the remainder of the tumor. Bars show median with 95% confidence interval. One-sample *t* test comparing fold enrichment to 1. (**H**) Line plot of immune cell fold enrichment in CIN hotspots across increasing hotspot thresholds. The heatmap indicates significance by a one-sample *t* test. (**I**) Representative immunofluorescence images of *CXCL8* RNAscope in CIN^high^ and CIN^low^ EAC tumors. Scale bars, 100 μm. (**J**) Tumor cell–intrinsic *CXCL8* mRNA expression in CIN^high^ versus CIN^low^ EAC tumors. Bars represent the means ± SEM. Significance determined by a Mann-Whitney *U* test. **P ≤ 0.01 and *P ≤ 0.05.

As our data supported a strong link between CIN-driven innate immune signaling with a distinct chemokine signaling pattern and increased myeloid infiltrate, we sought to further characterize the TME landscape of EAC tumors with ongoing CIN (Fig. 5A). Two tissue microarrays (TMAs) comprising 50 primary EAC tumor cores (24 treatment-naïve biopsy samples and 26 resections, containing 22 matched pre/postneoadjuvant chemotherapy samples) were profiled (patient clinical characteristics summarized in table S2). Treatment-naïve samples were first profiled using imaging mass cytometry (IMC), using a 32-color hyperplexed panel, with 16 samples suitable for detailed immunophenotyping (fig. S10, A to D, and table S3).

Correlating mIF-derived cGAS^+^ MN scores with immune cell abundances across tumors revealed an overall enrichment of intratumoral myeloid cells with increasing tumor CIN. Specific myeloid subsets exhibiting particularly strong associations with CIN included CD68^+^CD163^+^myeloperoxidase^+^ macrophages (MPO^+^ Mφ), a protumorigenic tumor-associated macrophage population (*38*), as well as tumor-associated neutrophils (CD15^+^HIF-1α^+^; Fig. 5B). Notably, assessment of tumor cell proliferation through Ki67 staining or proliferation-associated gene expression (E2F targets) revealed a moderate positive, albeit statistically insignificant, correlation with cGAS^+^ MN frequency, suggesting that elevated MN production is not solely attributable to higher cell division (fig. S10E).

To further characterize CIN-associated immune infiltrates in a broader cohort (comprising 38 tumors with assessable CIN scores), we stained our TMAs using a mIF panel that included Abs against myeloid cell markers (CD11c, CD11b, CD68, and CD206), as well as CD3, pCK, and DAPI (fig. S11, A to C). Observed patterns of immune marker positivity overlap, reflecting the phenotypically plastic and heterogeneous myeloid compartment, were used to classify stromal cells as macrophages (CD68^+^; Mφ), M1-like macrophages (CD68^+^CD11c^+^CD206^−^), M2-like macrophages (CD68^+^CD206^+^), dendritic cells (DCs; CD11c^+^CD68^−^), and tumor-associated neutrophils (Nφ; CD11b^+^CD68^−^; fig. S11, D to F). CD3^+^ cells were classified as T cells. Peripheral blood neutrophil counts correlated with intratumoral neutrophil infiltrate but not other immune cell infiltrate (fig. S11G), suggesting that intratumoral neutrophil inflammation tracks peripheral neutrophil inflammation.

Multiplex IF-derived immune cell abundances correlated closely with IMC-derived measurements (fig. S11H). Consistent with IMC results, in the broader mIF-stained TMA cohort, overall myeloid infiltration, as well as M2-like macrophage and tumor-associated neutrophil abundance, again showed significant associations with cGAS^+^ MN frequencies (Fig. 5, C and D).

To map immune cell infiltrates within CIN^high^ niches (“CIN hotspots”) for each tumor, we spatially aligned cGAS^+^ MN detections onto adjacent mIF immune panel–stained images and used a probability density function estimation approach to define CIN hotspots (Fig. 5E). We then quantified the relative proportion of infiltrated immune cells within CIN hotspots versus the remainder of the tumor (Fig. 5F). Tumor regions with high cGAS^+^ MN densities were proportionally enriched for macrophages and M2-like macrophages. In contrast, T cells and DCs appeared to be largely restricted from CIN^high^ regions (Fig. 5, G and H).

As these data suggested that cGAS^+^ MN–rich tumor regions were preferentially macrophage and myeloid cell infiltrated, we sought to confirm whether elevated cGAS^+^ MN frequencies coincided with enhanced CIN-associated myeloid-attracting chemokine expression in situ. To do so, we performed *CXCL8* RNA in situ hybridization (RNAscope) (*39*), measuring the extent of *CXCL8* expression in malignant and stromal regions of tumor cores (fig. S12, A and B). Across tumor cores, malignant cell expression of *CXCL8* was associated predominantly with cGAS^+^ MN frequencies (Fig. 5, H and I), as well as with intratumoral myeloid cell population (macrophages and neutrophils) infiltration (fig. S12C). In contrast, *CXCL8* expression in the stromal compartment was most significantly correlated with infiltrated neutrophil abundance, in keeping with its high reported expression in this cell population (fig. S12, D and E) (*40*, *41*). Together, these data indicate that IL-8 derived from CIN^high^ tumor cells results in an inflamed, myeloid-skewed TME typically associated with protumorigenic activity.

To evaluate the impact of IL-8 on myeloid cell recruitment in CIN settings, we leveraged our in vitro–engineered chronic CIN (isogenic CP-A cells)– and acute CIN (MPS1i)–driven cell line models using transwell migration assays to measure the migration of peripheral blood–derived human CD14^+^ monocytes—important precursors of tumor-infiltrating myeloid cells (*42*)—toward cell line–conditioned media (CM; fig. S13A). All three independent CIN^high^ CP-A p53^KO^dnMCAK clones exerted a significantly increased monocyte recruitment capacity over the matched genomically stable CP-A Cas9 clone (fig. S13B). Preincubation of monocytes with the CXCR1/2 inhibitor reparixin almost completely abrogated monocyte recruitment, demonstrating that the CIN-dependent recruitment capacity was largely dependent on the production of CXCR1/2 ligands, many of which we identified as major CIN-dependent chemokines. Similar results were observed using MPS1i-driven induction of CIN in OE33 and SK-GT-4 cells, where MPS1 inhibition resulted in monocyte recruitment in CM from cGAS-proficient, but not in cGAS^KO^, cells (fig. S13, C and D). The effect of cGAS^KO^ was partially recapitulated by CXCR1/2 inhibition, which did not abolish recruitment together, suggesting that additional cGAS–STING–dependent chemotactic factors beyond CXCR1/2 ligands are secreted by CIN^high^ cells.

### CIN reshapes the TME and drives therapeutic resistance in EAC

Leveraging the matched design of our TMA cohort [comprising 22 paired pre– and post–neoadjuvant treatment (NA Tx) samples], we next sought to assess how therapy reshapes the EAC immune landscape. This within-patient comparison revealed significant alterations restricted predominantly to myeloid populations, with decreased intratumoral neutrophil and M2-like macrophage frequencies, as well as increased M1-like macrophages following treatment (fig. S14A). Notably, high CIN in pretreatment tumors was associated with a significant posttreatment decline in T cell infiltration, suggesting that CIN may promote ongoing T cell exclusion and immunosuppression throughout treatment (fig. S14B).

As our results indicated that elevations in CIN were associated with myeloid-enriched, immunosuppressive and potentially therapy-resistant microenvironments, we investigated the relationship between the response to neoadjuvant chemotherapy (NA Tx) and immune features, including intratumoral immune infiltration, the extent of CIN and cGAS–STING protein expression across pretreatment tumors (fig. S14, C and D).

Expectedly, nonresponse to neoadjuvant chemotherapy [defined as tumor regression grades (TRGs) 4 and 5; also known as Mandard score] was associated with poor overall and recurrence-free survival (fig. S14E). In keeping with a previous report (*43*), increased T cell and DC infiltrate was associated with improved response to neoadjuvant chemotherapy (defined as TRGs 1 and 2; fig. S14F), whereas an increased intratumoral macrophage–to–T cell skew was associated with resistance (fig. S14G). However, neither cGAS^+^ MN burden nor cGAS or STING protein expression alone was significantly associated with response (fig. S14, H and I).

We next used a linear modeling approach to assess whether the extent of CIN exhibited response-specific associations with tumor immune features (fig. S14J). In contrast to NA Tx responders (R), CIN^high^ nonresponder tumors were marked by a decrease in STING protein levels in malignant cells, consistent with previously reported STING tachyphylaxis in CIN^high^ settings, associated with a rewired cGAS–STING pathway driving a tumor-supportive myeloid infiltrate (fig. S14, J and K) (*17*). Consistent with this, increased CIN was associated with an increased macrophage:T cell skew, specifically in nonresponder versus responder tumors (fig. S14, J and L). Pretreatment tumors from nonresponder patients were predominantly composed of CIN^high^, myeloid-dominated and CIN^high^STING^low^ tumors, features that were largely absent among responder patients (fig. S14, M and N).

**Fig. 6.**
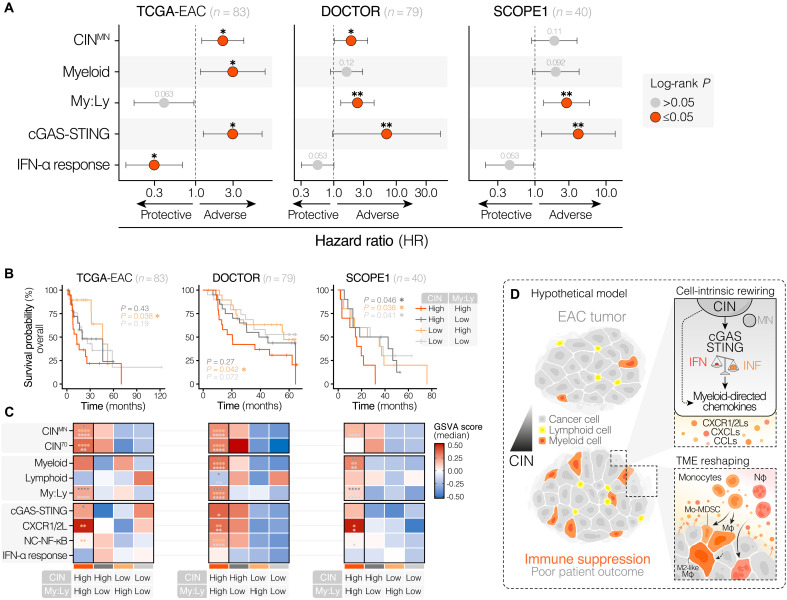
Chromosomally unstable, myeloid-dominated EAC tumors are associated with poor patient prognosis. (**A**) Forest plots of Cox proportional hazards models, showing the hazard ratio of high expression (determined using the maximal statistic approach) for indicated signatures in three independent EAC RNA-seq cohorts. Significance was determined using the log-rank test. (**B**) Kaplan-Meier curves showing the overall survival probability of human patients with EAC partitioned according to CIN^MN^ score (median stratification) and subsequent partitioning (median) of CIN^high^ and CIN^low^ groups based on the degree of myeloid cell enrichment. Myeloid cell enrichment was determined by computing the extent of myeloid–to–lymphoid cell skew (My:Ly) for each patient using snRNA-seq–derived immune cell type signatures. Significance was determined through pairwise comparisons between the CIN^high^, myeloid-dominated group and other groups using the log-rank test. (**C**) Heatmaps showing the median expression for indicated signatures among patient clusters of interest. Asterisks denote the significance, determined through an ANOVA with Tukey’s post hoc test, of the difference between the CIN^high^, myeloid-dominated cluster and other clusters (denoted by asterisk color). ****P ≤ 0.0001, ***P ≤ 0.001, **P ≤ 0.01, and *P ≤ 0.05. (**D**) Hypothetical model through which CIN drives cGAS–STING–dependent and cGAS–STING–independent intratumor myeloid cell inflammation.

To identify the role of CIN in clinical outcomes in EAC, we calculated CIN^MN^ scores derived from bulk RNA-seq data to stratify EAC tumors across three independent datasets [TCGA (*n* = 83) (*44*, *45*), DOCTOR (*n* = 79) (*46*), and SCOPE1 (*n* = 40) (*47*) trials]. On the basis of our results supporting myeloid:T cell enrichment as a significantly associated parameter with poor response to neoadjuvant chemotherapy, we derived a myeloid enrichment score measuring myeloid:lymphoid skew using gene signatures derived from our snRNA-seq data (table S1). Univariate Cox analyses across datasets supported the association of CIN^MN^ scores with worse pathological outcomes (Fig. 6A). Consistent with our findings, an increased myeloid:lymphoid skew and cGAS–STING mRNA abundance were also associated with clinical outcomes.

CIN^MN^-high, myeloid-dominated tumors were consistently characterized by elevated CXCR1/2 ligands, noncanonical NF-κB signaling, and cGAS–STING expression and exhibited significantly worse prognoses across the TCGA cohort and trial cohorts of patients with EAC treated with chemotherapy and chemoradiotherapy (Fig. 6, B and C) (*46*, *47*). Collectively, our results indicate that cGAS-dependent chemokine signaling in CIN^high^ EAC contributes to a profoundly immunosuppressive myeloid-rich TME, resulting in nonresponse to neoadjuvant therapy and poor clinical outcomes for patients with this aggressive cancer subtype (Fig. 6D).

## DISCUSSION

CIN and chronic inflammation are prevalent features of EAC, emerging early in disease progression and promoting the aggressive characteristics of this tumor. Here, we demonstrate that EAC cells retain some aspects of the cGAS–STING pathway, whose activation by CIN-associated cytoplasmic dsDNA converges on a prominent engagement of inflammatory signaling and the production of cytokines and chemokines acting on myeloid leukocytes. By coupling the scoring of cGAS^+^ MN, which we validated as correlating to CIN by complementary WGS of cells and tumors, with snRNA-seq and spatial mapping of human EAC tumors, we demonstrate that CIN^high^ tumors are associated with heightened inflammatory activity and a myeloid-enriched, immunosuppressive microenvironment.

Our data revealed the enrichment of negative regulators of type I IFN signaling, including up-regulation of USP18 (ubiquitin-specific peptidase 18), as well as IFI35 (IFN-induced protein 35), associated with CIN^high^ tumors. In contrast, we also observed that IFN-responsive genes, including STAT1, scaled with tumoral cGAS^+^ MN burden—in line with recent findings in genomically unstable breast cancer (*48*)—suggesting that enhanced STAT1 activation may impose type I IFN–centered selection pressures on CIN^high^ EAC. Notably, our ex vivo findings of STAT1, IFN-α, and IFN-γ response pathway enrichment with increasing CIN differed from in vitro results that indicated down-regulation of IFN response pathways with CIN. This can be partly accounted for by the complexity of the TME compared to a two-dimensional cell line model, with amplification of CIN-induced immune signaling resulting in a rewired TME. Our data are in keeping with recent reports that highlight potential tumor promotion in chronically IFN-active TMEs, whereby targeting of IFN or JAK/STAT pathways can sensitize tumors to immune checkpoint blockade (*49*–*52*). Whether this is of similar therapeutic potential in patients with CIN^high^ EAC remains to be elucidated.

Our findings contrast with reports of aneuploid tumors as “immune cold” (*53*, *54*). This has been attributed in part to the loss of chromosome 9p and the associated IFN gene cluster and, thus, loss of IFN and inflammatory signaling, as well as loss of antigen presentation machinery (*55*–*57*). However, we find sustained and enhanced inflammatory signaling with increased CIN in EAC, associated with an inflamed TME. While this could be a site-specific phenomenon, it may reflect that CIN^high^ and aneuploid tumors are distinct entities, with associated patterns of immune infiltrate and inflammatory signaling. Making this differentiation is crucial, as aneuploid tumors and tumors with ongoing CIN may not share overlapping therapeutic vulnerabilities. A challenge in measuring CIN has been the dynamic nature of this state, as a genomic snapshot of a CIN^high^ tumor resembles that of an aneuploid tumor. Therefore, measurement of cGAS^+^ MN may enable distinction of active CIN tumors compared to those in a steady state of aneuploidy, with inflammatory or immune-restricted TMEs, respectively.

To avoid imposing arbitrary CIN thresholds, we largely treated CIN as a continuous variable, enabling us to capture the full spectrum of CIN phenotypes and examine CIN dose-dependent biological and immune effects. However, while immunocytochemistry-based detection of MN holds promise as a robust and accessible biomarker for ongoing CIN (*58*), its broader implementation will require the establishment of reproducible scoring approaches and standardized quantitative thresholds (what constitutes “high CIN”?), as well as further demonstration of biological and prognostic relevance across diverse tissue contexts. Here, cGAS^+^ MN were scored as a proxy for the extent of cGAS–STING–activating chromosome segregation errors. However, recent reports have suggested that MN may not be the sole or potentially even the primary source of cGAS activation in response to CIN (*20*–*23*). CIN^high^ tumor cells may contain diverse nucleic acid species in their cytosol beyond micronucleated genomic DNA, including double-stranded RNA (*59*) and RNA–DNA hybrids (*60*), that may activate cGAS or engage other innate immune cytosolic sensors such as MDA5 (melanoma differentiation–associated protein 5) or RIG-I (retinoic acid–inducible gene I protein). The exact biochemical nature of the CIN-associated cGAS agonist remains to be established, although it is most likely closely linked to MN formation.

The chemokine IL-8 (*CXCL8*) is heavily implicated in promoting tumor progression via direct impact on cancer cell proliferation as well as through the recruitment of myeloid-derived suppressor cells, tumor-associated neutrophils, and macrophages (*61*). Elevated circulating IL-8 in patients receiving immune checkpoint blockade has been reportedly associated with poorer outcomes (*62*, *63*). However, the source of IL-8 has been unclear. Here, we identify CIN-driven cGAS activation as resulting in increased *CXCL8* expression in CIN^high^ cancers, thereby accounting for the intratumoral myeloid infiltrate observed. This is consistent with in vivo studies that have reported that the murine homolog *Cxcl1* is enriched in CIN^high^ tumor models (*17*, *25*). Targeting the receptors for IL-8, particularly CXCR2, has been explored in early phase clinical trials in prostate cancer (*64*) and hepatocellular cancer (*65*). Initial responses are promising, although peripheral neutropenia and risk of bacterial sepsis suggest that tumor-specific targeting may be required. Mice do not express *CXCL8*, and although homologs *Cxcl1* and *Cxcl2* recapitulate some functions, these do not perfectly mirror human *CXCL8* with nonredundant roles reported (*66*). Species-restricted biology of the IL-8-CXCR1/2 axis therefore requires near-human models to accurately understand its role in tumor growth and therapeutic response (*67*).

Locally and systemically administered STING agonists are under clinical development in combination with immune checkpoint inhibitors (*68*). Early results of intratumorally administered STING agonists have not demonstrated broad efficacy. A potential explanation for the discrepancies observed between murine models and patient responses could be that STING expressed in tumor cells is poised to induce a type of inflammation that is immunosuppressive and protumor. CIN^high^ tumors with chronic cGAS–STING activation may therefore be less likely to benefit from STING agonist strategies.

The poor clinical outcomes experienced by patients with CIN^high^ tumors are unlikely to be rescued by the advent of immune checkpoint inhibitors in EAC (*69*, *70*) as CIN is associated with resistance to immunotherapy (*71*). Therefore, innovative therapeutic interventions are urgently required to transform care for patients with CIN^high^ cancers. Our findings explain the counterintuitive maintenance and heightened expression of cGAS and STING in these aggressive tumors and suggest that therapeutic approaches that disrupt the cGAS-driven protumor inflammatory axis in CIN^high^ tumors may be effective strategies.

## METHODS

### Cell culture

ESO-26, ESO-51, OACM5.1C, OE19, OE33, and SK-GT-4 cell lines were cultured in RPMI 1640, FLO-1 EAC, and human embryonic kidney (HEK) 293T cells were cultured in Dulbecco’s modified Eagle’s medium; KYAE-1 cells were cultured in RPMI 1640:Hams F12 (1:1 ratio); and hTERT-immortalized RPE-1 cells were cultured in Dulbecco’s modified Eagle’s medium:Ham’s F-12. Media were supplemented with 10% fetal bovine serum (FBS) and penicillin and streptomycin (100 U/ml). BE-derived hTERT-immortalized CP-A (American Type Culture Collection, KR-42421) and CP-C (American Type Culture Collection, CRL-4029) cells (*34*, *72*) were cultured in MCDB-153 supplemented with hydrocortisone (0.4 μg/ml), recombinant human epidermal growth factor (20 ng/ml), cholera toxin (8.4 μg/ml), adenine (20 mg/ml), bovine pituitary extract (140 μg/ml), 1× insulin-transferrin-selenium, 4 mM l-glutamine, penicillin and streptomycin (200 U/ml), and 5% FBS. All cell lines were maintained in a 37°C humidified incubator at 5% CO_2_ and routinely tested negative for mycoplasma contamination.

### Vector engineering and stable cell line generation

To generate cell lines with *TP53*, *CDKN2A*, and *CGAS* KO, target sequences were cloned into pL-CRISPR.SFFV.eGFP (Addgene, plasmid no. 57827), pL-CRISPR.SFFV.tRFP (Addgene, plasmid no. 57826), or lentiCRISPR v2 (Addgene, plasmid no. 52961) transfer vector backbones according to the lentiCRISPRv2-Puro target guide sequence cloning protocol (*73*). Single-guide RNAs (sgRNAs) are summarized in table S4. Plasmids were transformed into One Shot Stbl3 Chemically Competent *Escherichia coli* (Invitrogen), and successfully transformed clones were selected from LB-agar ampicillin (100 mg/ml) plates. To confirm successful sgRNA integration, plasmids were sequenced by Sanger sequencing (TubeSeq, Eurofins Scientific).

To obtain recombinant lentivirus for transduction, ~3.0 × 10^6^ HEK293T cells were transfected with Lipofectamine 2000 (Invitrogen, 11668027) and Opti-MEM, supplemented with 5 μg of the appropriate transfer vector, 2.5 μg of pCMV-VSV-G (Addgene, no. 8454) envelope vector, and 2.5 μg of pCMV-delta R8.2 (Addgene, no. 12263) packaging vector. Successful transfection of HEK293T cells with CRISPR.SFFV.eGFP (Addgene, plasmid no. 57827) and CRISPR.SFFV.tRFP (Addgene, plasmid no. 57826) vectors was confirmed by epifluorescence microscopy. Lentivirus was used to transduce CP-A or EAC cells in the presence of polybrene (5 μg/ml; Sigma-Aldrich). Successfully transduced cells were selected via puromycin (1.5 μg/ml) treatment [for lentiCRISPR v2 (Addgene, plasmid no. 52961) and pLV-CMV-dnMCAK (Addgene, plasmid no. 205993) transduction] or flow cytometric single-cell sorting of GFP^+^ or GFP^+^RFP^+^ cells using a BD FACSAria Fusion Flow Cytometer (BD Biosciences; for pL-CRISPR.SFFV.eGFP and pL-CRISPR.SFFV.tRFP transductions). Single-cell clones from puromycin-selected mixed populations were obtained through limiting dilution in 96-well plates.

CP-A–derived single-cell clones (Cas9 control, p53^KO^, p53p16^DKO^, and p53^KO^dnMCAK) were screened by amplicon sequencing to confirm successful disruption of sgRNA target sites. PCR primers are detailed in table S5.

### Flow cytometry

To estimate the degree of green fluorescent protein (GFP) positivity in pL.CRISPR.SFFV.eGFP (Cas9 control)– and pL.CRISPR.SFFV.eGFP.sgTP53 (p53^KO^)–transduced CP-A cells, cells were trypsinized, washed twice by centrifugation (5 min at 300*g*), and resuspended in phosphate-buffered saline (PBS). Data acquisition was performed using an Attune NxT Flow Cytometer (Thermo Fisher Scientific). Side scatter area (SSC-A)/forward scatter area (FSC-A) and forward scatter height (FSC-A/FSC-H) gates were set to exclude cell debris and cell doublets, respectively. RL1 and BL1 gates were set to select enhanced GFP (eGFP)–positive cells. Data analysis was performed using FlowJo (version 10.4) software.

### Immunoblotting

#### Cell lysate preparation

Cells were lysed in radioimmunoprecipitation assay lysis buffer (Thermo Fisher Scientific) supplemented with protease inhibitor cocktail (Roche, CO-RO) and phosphatase inhibitor (Roche, PHOSS-RO). Cell lysates were sonicated for 20 s at a 10% amplitude, and resulting homogenates were cleared by centrifugation at 16,000*g* for 15 min at 4°C. Protein concentrations were derived using the BCA (bicinchoninic acid) Protein Assay (Thermo Fisher Scientific).

#### Western blotting

Lysates were boiled for 5 min at 90°C in 4× Laemmli Sample Buffer (Bio-Rad) supplemented with 355 mM 2-mercaptoethanol. Equal amounts of protein were resolved by SDS–polyacrylamide gel electrophoresis on 4 to 20% polyacrylamide gels and transferred onto 0.2-μm nitrocellulose membranes. Membranes were blocked in 3% bovine serum albumin (BSA) in tris-buffered saline-Tween [50 mM tris-HCl (pH 7.5), 50 mM NaCl, and 0.1% Tween 20] for 1 hour at room temperature (RT) and probed by overnight incubation in primary Ab–blocking solution at 4°C. For bound primary Ab detection, membranes were incubated for 1 hour at RT in blocking buffer solution supplemented with either secondary anti-mouse (Cell Signaling Technology, no. 7076) or anti-rabbit (Cell Signaling Technology, no. 7074) streptavidin-conjugated horseradish peroxidase Ab (1:1000). Membranes were incubated in Immobilon Western horseradish peroxidase substrate (Sigma-Aldrich) for enhanced chemiluminescence–based protein detection and imaged using the ChemiDoc MP (Bio-Rad) or iBright FL1500 (Thermo Fisher Scientific) systems. Resulting images were analyzed using Image Lab Software 5.0 (Bio-Rad). Primary Abs are detailed in table S6.

### Immunofluorescence staining

#### Sample seeding

Adherent cells were seeded on glass coverslips. Suspension cells underwent treatments in suspension and were seeded onto poly-l/d-lysine–coated glass coverslips 6 hours before treatment end points. All cells were allowed to reach 70 to 90% confluence before sample collection.

#### Immunostaining

Cells were fixed with paraformaldehyde for 12 min at RT and permeabilized for 5 min in 0.5% Triton X-100, followed by blocking in 2.5% BSA in 0.05% PBS-Tween for 1 hour at RT. For binding of primary Ab, coverslips were inverted onto 75-μl drops of primary anti-cGAS Ab solution (1:200; Cell Signaling Technology, no. 15102) in 2.5% BSA in PBS-Tween and incubated overnight at 4°C. Primary Abs were labeled by incubation in an Alexa Fluor 488–conjugated goat anti-rabbit secondary Ab (Thermo Fisher Scientific, no. R37116) in blocking solution for 1 hour at RT. Cells were counterstained for DNA by incubation in Hoechst (5 μg/ml) for 15 min and mounted with Prolong Gold Antifade Mountant (Thermo Fisher Scientific).

#### Image acquisition, processing, and analysis

Images were acquired with a 63× oil immersion objective on an Andor Dragonfly spinning disc confocal microscope (Oxford Instruments) or with a 40× objective on an Olympus SpinSR SoRa spinning disc confocal microscope (Olympus Life Science). Images were acquired as *Z*-stacks (3 to 11 slices) taken at 1-μm intervals. Maximum intensity–projected images were processed and analyzed using the Fiji image processing package of ImageJ software (*74*).Custom Fiji macros were used to count nuclei and guide manual counts for MN and cGAS^+^ MN. CGAS^+^ chromatin bridges were scored manually.

### RT-qPCR

RNA isolation was performed using the RNeasy Plus Mini Kit (Qiagen), and complementary DNA (cDNA) was derived using the SuperScript III First-Strand Synthesis SuperMix (Thermo Fisher Scientific, 11752050) or HiScript IV SuperMix (Vazyme, R423-01). Quantitative RT-PCR was performed using Fast SYBR Green Mastermix (Applied Biosystems, 4385618) or Taq Pro Universal SYBR qPCR Master Mix (Vazyme, Q712-03) on a StepOnePlus Real-Time PCR System (Applied Biosystems). Relative abundance of mRNA was determined through the 2^−ΔΔCT^ method (*75*) using *18S* as the reference gene. Primer pair sequences are listed in table S7.

### 2′3′-cGAMP stimulation

EAC cells were seeded at a density of 5.0 × 10^5^ cells per well in six-well plates and allowed to adhere for 24 hours. Cells were then transfected with 2′3′-cGAMP (2 μg/ml; InvivoGen) or cGAMP diluent (sterile diethyl pyrocarbonate–treated water) with Lipofectamine 2000 (2 μl/ml; Invitrogen) in Opti-MEM media for 6 hours. Before transfection, 2′3′-cGAMP/diluent:lipofectamine transfection mixtures were incubated at RT for 20 min to facilitate transfection complex formation.

### 2′3′-cGAMP ELISA

Intracellular and extracellular 2′3′-cGAMP quantification was largely carried out as previously described (*16*). Cells were seeded in 100-mm culture dishes and allowed to grow to a confluency of 80 to 90% before changing the media to reduced serum and phenol red–free Opti-MEM media (Thermo Fisher Scientific). Cells were treated with 1 μM MPS1i reversine or dimethyl sulfoxide (DMSO) 48 hours before medium exchange. For quantification of cGAMP levels following dsDNA-driven cGAS stimulation, cells were transfected with G_3_-YSD Y-form DNA (2.5 μg/ml; InvivoGen, tlrl-ydna) using Lipofectamine 2000 (2 μl/ml; Invitrogen) in Opti-MEM media for 24 hours.

For extracellular cGAMP, conditioned media were collected 24 hours following medium change and centrifuged at 600*g* at 4°C for 15 min. For intracellular cGAMP, adherent cells were collected and counted. Cell suspensions were pelleted for 15 min at 600*g* at 4°C, lysed in radioimmunoprecipitation assay lysis buffer, and sonicated for 10 s at a 10% amplitude. Resulting homogenates were cleared by centrifugation, and protein concentrations were derived by the BCA assay. For intracellular cGAMP quantification, protein concentrations were adjusted to 0.5 to 1 mg/ml (kept consistent within experiments). Conditioned media for extracellular cGAMP quantification were assayed directly. cGAMP ELISA was performed using the 2′3′-Cyclic GAMP Competitive ELISA Kit (Invitrogen). 2′3′-cGAMP concentrations of cell supernatants were normalized to absolute cell counts for each sample.

### IL-8 ELISA

For quantification of IL-8 concentrations of cell culture supernatants, OE33, SK-GT-4, and CP-A cells were seeded at a density of 3.0 × 10^5^ or 5.0 × 10^5^ cells in 100-mm cell culture dishes. After 24 hours, cell media were either replaced with fresh media or, where indicated, replaced with 1 μM reversine- or DMSO-treated media for 24 hours, followed by drug washout and replacement with fresh media. Cell culture media were conditioned for 48 hours, collected, and cleared by centrifugation at 1000*g* for 10 min at 4°C. IL-8 ELISA was performed using the human IL-8 ELISA kit (Thermo Fisher Scientific).

### Viability assay

Cells were plated in 96-well plates at a density of 1500 or 2000 cells per well (EAC cells and CP-A lines, respectively) to ensure that mock-treated cells reached ~90% confluency at the assay end point. After 24 hours, cells were treated with puromycin, Nutlin-3a (Selleck Chemicals), or reversine. Cell viability was assessed using the Cell Counting Kit 8 (CCK8; Abcam). WST-8 solution was added to wells 4 hours before treatment end point. Cell viability was determined by measuring absorbance at 460 nm and normalizing to the average absorbance of mock-treated wells.

### RNA sequencing

#### RNA-seq and data preprocessing

EAC and CP-A cells were seeded at a density of 5.0 × 10^5^ cells in 100-mm cell culture dishes. After 24 hours, cell culture media were replaced with either fresh media or 0.5 μM reversine- or DMSO-treated media for CP-A and EAC cells, respectively, and incubated for an additional 48 hours. RNA was isolated using the RNeasy Plus Mini Kit (Qiagen), and sample quality was assessed using the 5400 Fragment Analyzer System (Agilent). Paired-end 150–base pair (bp) sequencing of cDNA libraries was performed on the Illumina NovaSeq6000 platform. Reads were mapped to the human reference genome GRCh38 using HISAT2 (version 2.0.5) (*76*). The resulting alignments were summarized and quantified at the gene level using featureCounts (version 1.5.0) to obtain read counts for downstream differential expression analysis (*77*).

#### Identification of cGAS-dependent CIN-induced genes

To assess MPS1i treatment–induced gene expression changes in cGAS-proficient and cGAS^KO^ EAC cells, batch effects were corrected using the “ComBat” function from the sva R package (version 3.36.0) (*78*). Genes were filtered to retain those with a total read count of ≥5 counts across all samples and nonzero expression in ≥4 samples. Filtered datasets were analyzed for differential expression using DESeq2 (version 1.42.0) (*79*), and significance was tested using the default Wald test, corrected for multiple testing via the Benjamini-Hochberg method, between *n* = 3 biological replicates of MPS1i- and DMSO-treated samples. Significantly differentially expressed genes were identified using an adjusted *P* ≤ 0.01 cutoff.

Genes whose MPS1i-induced differential expression was cGAS-dependent were identified by paired *t* tests, comparing the log_2_ fold change (FC) of significantly differentially expressed genes between MPS1i- and DMSO-treated samples in cGAS-proficient (Cas9) versus cGAS^KO^ cells. Genes that were significantly up-regulated [adjusted *P* ≤ 0.01, log_2_(FC) > 0] in MPS1i-treated cGAS-proficient cells with a significantly reduced MPS1i-induced up-regulation (paired *t* test, *P* ≤ 0.05; paired by batch number) in cGAS^KO^ cells were classified as cGAS-dependent MPS1i-induced genes. Conversely, genes that were significantly down-regulated [adjusted *P* ≤ 0.01, log_2_(FC) < 0] in MPS1i-treated cGAS-proficient cells, with a significantly less pronounced down-regulation (*P* ≤ 0.05) in cGAS^KO^ cells, were considered cGAS-dependent MPS1i-suppressed genes.

#### Identification of cGAS^+^ MN–correlated genes

To identify genes whose expression correlated with the baseline cGAS^+^ MN burden of cell lines in our isogenic CP-A cell line model (Cas9 control, p53^KO^, p53p16^DKO^, and p53^KO^dnMCAK lines), gene-level counts were filtered to retain genes with a cumulative read count of ≥50 counts across all (*n* = 30) samples and nonzero expression in ≥4 samples. Filtered counts were then normalized using variance-stabilizing transformation from the DESeq2 package.

For each gene, a linear mixed-effects model was fitted using the “lm” function of the nlme R package (version 3.1-165) (*80*) to assess the relationship between gene expression and cGAS^+^ MN burden (the number of baseline cGAS^+^ MN per 100 cells; i.e., cGAS^+^ MN %). The model included the average cGAS^+^ MN burden (*n* = 3, as determined by IF) for each cell line as a fixed effect and incorporated random effects attributable to different clones (*n* = 3 independent cell clones) and experimental batches (*n* = 3 biological replicates), enabling identification of genes whose expression significantly scaled with cGAS^+^ MN burden while accounting for confounding effects stemming from clone and batch variability.

Specifically, the model was defined as follows

$${\text{Expression}}_{\left(\text{gene}\;X\right)}∼{\text{average cGAS}}^{+}\;\text{MN}+\left(1|\text{Clone}/\text{Batch}\right)$$

For each gene, the *P* value [analysis of variance (ANOVA); indicating the statistical significance of the association between expression and cGAS^+^ MN burden] and slope (magnitude change in gene expression per unit increase in cGAS^+^ MN burden) of the cGAS^+^ MN effect were extracted from the model output and used to identify hits whose expression was most strongly associated with MN burden.

### Functional enrichment analyses

#### Gene set enrichment analysis

GSEA was performed using the fgsea (version 1.28.0) package (*81*) in R, using the Hallmark and Canonical Pathways [composed of Reactome, Kyoto Encyclopedia of Genes and Genomes (KEGG), Pathway Interaction Database (PID), WikiPathways, and BioCarta databases] collections from the Molecular Signatures Database (MSigDB) (*82*), as well as Gene Ontology Biological Processes (GO:BP) database gene sets (*83*). Gene set collections were retrieved using the msigdbr (version 7.5.1) package (*84*).

To evaluate the broader distribution of enrichment for gene sets related to cell cycle, DNA damage repair (DDR), IFN response, and inflammatory response pathways in GSEA outputs, gene sets were classified on the basis of character strings in their assigned pathway names. Gene sets identified as belonging to cell cycle, DDR, IFN, or INF pathway groups were compiled into custom lists (table S8) and analyzed for enrichment through GSEA using rank metrics for gene sets computed from the original GSEA outputs.

#### Overrepresentation analysis

Overrepresentation analysis was performed using the “gost” function of the gprofiler2 R package (version 0.2.3) (*85*), focusing on GO:BP and Reactome database pathways. Enrichment probabilities were computed using the default one-sided Fisher’s exact test corrected for multiple testing via the Benjamini-Hochberg method. Database terms mapping to >2000 genes were excluded from the final analysis outputs, as they were considered too broad to provide meaningful insights.

### Single-cell WGS

To evaluate the karyotypes of our isogenic CP-A cell line model, ~1 × 10^6^ cells of each genotype and clone (Pa, Cas9 control, p53^KO^, p53p16^DKO^, and p53^KO^dnMCAK; clones 1, 2, and 3) were profiled using shallow scWGS (Research Sequencing and iPSC/CRISPR Facility, ERIBA, University Medical Centre Groningen, University of Groningen). Isolation and staining of nuclei, as well as nucleus sorting and library preparation, were performed as previously described (*86*), with small variations. Briefly, 24 single nuclei per cell line were dry sorted into 96-well plates. Overnight rehydration in thermolabile proteinase K was performed, and proteolysis started. MNase Digestion, AMPure Bead Cleanup and End Repair, and A-Tailing steps were replaced in favor of the Fragmentase enzyme (NEBNext Ultra II FS DNA Library Prep Kit, New England Biolabs). Subsequent library preparation steps were unchanged.

Libraries were sequenced on a NextSeq2000 (Illumina) P1-100c (1 × 10^6^ reads per single cell) sequencer using a 77-bp read-length and 11-bp dual index read configuration. Resulting FASTQ files were mapped to the human reference genome GRCh38 using the Burrows–Wheeler aligner (*87*). The aligned read data (BAM files) were analyzed using the AneuFinder algorithm (*35*). Following GC correction and blacklisting of artifact-prone regions (i.e., extremely low or high coverage in control samples), libraries were analyzed using the Dnacopy and Edivisive copy number calling algorithms with variable width bins (average bin size, 1 Mb; step size, 500 kb). Breakpoints were refined using refine.breakpoints = TRUE. Analyses were carried out using a euploid reference, as described previously (*86*).

Samples corresponding to p53^KO^dnMCAK clone 1 and p53^KO^ clone 2 were reanalyzed with the developer version of AneuFinder [version 1.7.4; available at https://github.com/ataudt/aneufinder (*35*)] using a minimum ground ploidy of 3 or 3.5 (min.ground.ploidy = 3.0 or 3.5) and a maximum ground ploidy of 4 or 4.5 (max.ground.ploidy = 4.0 or 4.5), respectively. As some of the libraries of these two samples gave differing results (copy numbers too low compared to other libraries; less optimal fits), results were curated by requiring a minimum concordance of 90% between the results of the two algorithms.

Libraries with on average <10 reads per bin and per chromosome copy were discarded. The aneuploidy score of each bin was calculated as the absolute difference between the observed copy number and the expected copy number when euploid. The score for each library was calculated as the weighted average of all the bins (size of the bin as weight), and the sample scores were calculated as the average of the scores of all libraries. The heterogeneity score of each bin was calculated as the proportion of pairwise comparisons (cell 1 versus cell 2, cell 1 versus cell 3, etc.) that showed a difference in copy number (e.g., cell 1: 2-somy; and cell 2: 3-somy). The heterogeneity score of each sample was calculated as the weighted average of all the bin scores using the size of the bin as weight.

### Whole-genome sequencing

DNA was extracted from patient-matched tumor and normal (buffy coat) samples using the DNeasy Blood and Tissue Kit (Qiagen) according to the manufacturer’s protocol. DNA samples were quantified using the Qubit 2.0 Fluorometer (Life Technologies), and DNA integrity was checked using the TapeStation 4200 (Agilent). The NEBNext Ultra II DNA Library Prep kit (New England Biolabs) was used for DNA library preparation following the manufacturer’s recommendations. Briefly, genomic DNA was fragmented by acoustic shearing using a Covaris instrument. Fragmented DNA was end repaired and adenylated. Adapters were ligated after adenylation of the 3′ ends, followed by enrichment by limited cycle PCR. Resulting adapter-ligated DNA libraries were cleaned up, validated using the Agilent TapeStation, and quantified using a Qubit 2.0 Fluorometer.

Short-read WGS of multiplexed libraries was performed on the Illumina NovaSeq platform with a paired-end 150-bp read configuration at a target coverage of 60× and 30× for tumor and normal samples, respectively. Resulting raw sequencing data (.bcl) files were converted to FASTQ format and demultiplexed using bcl2fastq software (Illumina).

Reference-based mapping on raw sequencing data was conducted by aligning FASTQ files to the GRChg38 reference genome using BWA-mem (version 0.717) (*87*), generating BAM files for downstream analysis. Resulting BAM files were subsequently sorted, indexed, and marked for duplicates using Picard (version 3.1.0) (*88*) and Samtools (version 1.15) (*89*).

SVs, tumor purity, and ploidy were called using the LINX pipeline (HMF tools, version 3.9) (*90*, *91*). SVs were categorized by PURPLE as deletions, duplications, inversions, or break-ends and were filtered to remove failed calls.

Copy number analysis was performed using ASCAT (version 3.1.2), JaBbA (version 1.0), and PURPLE. JaBbA (*92*) was used to visualize copy number alterations and SVs for each tumor. The percentage of the genome altered was calculated using major copy number per segment data from ASCAT by determining the number of megabases that were amplified, deleted, or unaffected (copy number, 2). Amplifications were defined by a copy number >2 and deletions by a copy number <2. The percentage of the genome amplified was calculated using PURPLE when the copy number exceeded 2.

### Single-nucleus RNA-seq

#### Tissue processing

To enable tissue-sparing extraction of nuclei for snRNA-seq from clinical-grade frozen tissue specimens, we optimized a previously described adaptation of the salt-tris (ST)–based extraction method (*93*, *94*) for EAC tissue samples. Clinical samples were collected under the Oxford Radcliffe Biobank–approved study 21/A093, with informed consent for research use obtained from patients treated at Oxford University Hospitals Trust.

Frozen tissue specimens were embedded in the optimal cutting temperature compound on dry ice and mounted on the sample holder of a LeicaCM1950 cryostat (Leica Biosystems) set to −20°C. Between 40 and 60 20-μm tissue scrolls were cut per tissue and stored at −80°C until further sample processing.

All subsequent sample processing steps were performed on wet ice using ice-cold buffers and centrifuges set to 4°C, unless stated otherwise. For extraction of nuclei, sample tubes were moved from dry to wet ice and allowed to equilibrate for 30 s. Next, 5 ml of sterile PBS was added to tubes, and samples were inverted three times to dissolve the optimal cutting temperature compound, followed by centrifugation at 300*g* for 2 min. Resulting tissue pellets were resuspended in 2 ml of ST buffer (146 mM NaCl, 10 mM tris-HCl, pH 7.5, 1 mM CaCl_2_, and 21 mM MgCl_2_ in diethyl pyrocarbonate–treated water) supplemented with 0.03% Tween 20 and 0.1% BSA. Cell suspensions were pipetted vigorously to mechanically disrupt the tissue and incubated on ice for 7.5 min, repeating the pipetting step every 2.5 min. Following the incubation period, homogenates were quenched by adding 1 ml of ST buffer with RNaseOUT ribonuclease inhibitor (40 U/ml; Thermo Fisher Scientific) and filtered twice through 700-μm filters. Remnants of dissociated tissue in sample tubes were rinsed with 3 ml of ST buffer with a ribonuclease inhibitor and filtered to enhance nucleus yields. Nucleus suspensions were then centrifuged at 500*g* for 5 min and resuspended in 1 ml of ST buffer. To determine the concentration and degree of dissociation of nuclei in the samples, nuclei were stained with Hoechst and counted using an EVOS M5000 benchtop fluorescence microscope (Thermo Fisher Scientific).

#### snRNA library preparation

Fourteen thousand nuclei (with the exception of sample no. 5, for which ~7600 nuclei were loaded) were loaded in ST buffer on a Chromium X using the Chromium Next GEM Single Cell 5′ Kit v2 (10x Genomics, PN-1000263) following the Chromium Next GEM Single Cell 5′ v2 (Dual Index) user guide (10x Genomics, CG000331 Rev E). gel beads in emulsion (GEMs) underwent reverse transcription, cleanup using Dynabeads MyOne Silane beads (Thermo Fisher Scientific), and amplification. To account for the lower abundance of RNA in nuclei versus whole cells, an additional PCR amplification cycle was added (14 total cycles). Construction of final gene expression (GEX) libraries was performed using the Library Construction Kit (10x Genomics, PN-1000190) and Dual Index Kit TT set A (10x Genomics, PN-1000215) according to the user guide. The fragment size distribution of cDNA and final sequencing-ready GEX libraries was assessed using a TapeStation 2200 system (Agilent) with TapeStation D5000 reagents. The concentrations of final GEX libraries were determined using a Qubit fluorometer (Life Technologies).

#### snRNA library sequencing

GEX libraries were sequenced on the Illumina NovaSeq X Plus platform with a paired-end 150-bp read configuration, covering ≥20,000 read pairs per cell. The sequencing run was set up with a 26-10-10-90–cycle configuration, consisting of 26 cycles for read 1, 10 cycles for the i7 index, 10 cycles on the i5 index, and 90 cycles for read 2. A 2% PhiX spike-in was included to monitor sequencing quality and provide an internal control.

#### Single-cell gene expression matrices

Generation of single-cell expression matrices, background noise removal, quality control, and filtering were performed according to a 10x Genomics Cell Ranger workflow–based analysis pipeline, as described previously (*94*). Demultiplexed FASTQ files from snRNA-seq reads were aligned to the human GRCh38 reference genome, and gene counts were quantified using the Cell Ranger (version 6.0.0, 10x Genomics) “count” function. As nuclear mRNA is highly intronic, introns were included in the analysis.

#### Background noise removal

Technical ambient RNA and empty droplets were removed from gene expression matrices using the “remove-background” function of CellBender (version 0.2.0) (*95*) on Cell Ranger–generated “raw_feature_bc_matrix.h5” files. The “Estimated Number of Cells” parameter inferred by Cell Ranger was used to define the “expected-cells” for extraction. The “total-droplets-included” parameter was adjusted to the midpoint of plateaus in the barcode-rank plot on the basis of visual inspection.

#### Quality control and filtering

Expression matrices were processed individually in R using the Seurat package (version 4.2.74) (*96*, *97*). Retained cells had detected genes ranging between 200 and 9000, unique molecular identifier (UMI) counts between 800 and 40,000, and mitochondrial reads <15%. Doublets were removed using Scrublet version 0.2.1 (*98*).

Filtered gene-barcode matrices were then normalized using the Seurat “NormalizeData” function and “LogNormalize” method. Variable genes were identified by applying the “vst” method in the “FindVariableFeatures” function, selecting the top 2000 variable genes. Next, gene expression matrices were scaled and centered using the “ScaleData” function. Dimensionality reduction was performed using principal components analysis (PCA) and uniform manifold approximation and projection (UMAP) using the top 30 principal components.

#### Single-cell cluster curation

Preprocessed data were normalized using “SCTransform,” followed by PCA dimensionality reduction. The resulting top 30 SCT principal components were used to integrate data across multiple samples using Harmony (version 1.2.0) (*99*), setting sample labels as grouping variables to enable correction for inherent batch effects. Integrated Harmony embeddings were then used to recompute the nearest neighbors and construct a shared nearest neighbors graph. Clustering of the shared nearest neighbors was performed using the “FindClusters” Seurat function (with res = 0.35) to identify cell clusters reflecting distinct cell types. Visualization of clusters was performed through UMAP using the top 30 principal components from Harmony-integrated PCA dimensionality reduction. Cluster identities were assigned by identifying differentially expressed genes between clusters using a variation of the negative binomial exact test on the basis of the sSeq method (*100*) implemented in the Cell Ranger pipeline. Resulting cluster markers were cross-referenced with the published literature to determine cell type identities, and clusters with similar functional marker genes were manually curated to assign high-level cluster identities. Differential gene expression (DGE) analysis of curated clusters yielded high-level cluster markers and was used to perform GSEA using –log_10_(adjusted *P* value) × sign[log_2_(FC)] from DGE outputs as gene-ranking metrics, enabling the assessment of cluster-level pathway enrichment.

#### Identification of cell type–specific cGAS^+^ MN–correlated genes

To enable the assessment of cell type–specific gene expression with tumoral cGAS^+^ MN burdens across samples, a pseudobulking approach was used to infer the relative cell type–specific expression level of genes across samples. Unnormalized RNA counts for each sample were aggregated on a per-cluster basis using the “AggregateExpression” function of the Seurat package. Aggregated expression matrices for each cell type were then normalized across samples using DESeq2. Expression values were then iteratively correlated with mIF-derived tumoral cGAS^+^ MN scores, yielding a Pearson correlation coefficient (*R*_p_) and *P* value. To infer the degree of pathway-level scaling with cGAS^+^ MN, GSEA was performed using the *R*_p_ for each gene as the rank metric.

#### Differential neighborhood abundance testing

To quantify differential cell state abundances between CIN^high^ and CIN^low^ tumors, we used the R package Milo (*101*), which models local variations in cellular composition using graph-based neighborhoods, using default parameters. Each neighborhood comprised an index cell and its surrounding cells in a *k*-nearest neighbor graph, representing discrete cell states within the dataset. For each predicted neighborhood, hypothesis testing between the compared groups was performed to identify differentially abundant cell states while controlling for false discovery rate (FDR). The number of cells in each neighborhood was counted on a per-sample basis to perform differential abundance testing. To correct for multiple hypothesis testing, a weighted FDR was used, accounting for the spatial overlap between neighborhoods.

To quantify differential cell state abundances between CIN^high^ and CIN^low^ tumors, we used the R package Milo (version 2.2) (*101*), which models local variations in cellular composition using graph-based neighborhoods, using default parameters. Each neighborhood comprised an index cell and its surrounding cells in a *k*-nearest neighbor graph, representing discrete cell states within the dataset. For each predicted neighborhood, hypothesis testing between the compared groups was performed to identify differentially abundant cell states while controlling for FDR. The number of cells in each neighborhood was counted on a per-sample basis to perform differential abundance testing. To correct for multiple hypothesis testing, a weighted FDR was used, accounting for the spatial overlap between neighborhoods. To prevent bias in weighted mean estimation, myeloid neighborhoods with log_2_FC < −3 were excluded following visual inspection of log_2_FC distributions, as high-confidence outliers disproportionately affected the analysis.

#### Myeloid and lymphoid cell signature development

To define EAC-associated intratumoral myeloid and lymphoid cell gene signatures, pairwise DGE analyses were performed between all curated subcompartment clusters using Cell Ranger’s implementation of the sSeq method. The top 50 (ranked by significance) genes exclusively significantly up-regulated in either myeloid or lymphoid subcompartments were defined as myeloid or lymphoid gene signatures, respectively, and are detailed in table S1.

### Multiplex immunofluorescence

#### TMA construction

The TMA samples were accessed from the Oxford Radcliffe Biobank (study number 20/A144), cut at the Oxford Centre for Histopathology Research, annotated and reviewed by a gastrointestinal histopathologist (A.E.), and assembled by the Translational Histopathology Laboratory (Oxford).

#### Multiplex immunostaining

mIF of human EAC biopsies was carried out on 4-μm FFPE sections on a Leica Bond RXm Autostainer (Leica Biosystems) using the Opal multiplex immunohistochemistry system (Akoya Biosciences). Slides were baked and dewaxed using a BOND dewax solution (Leica Biosystems, AR9222). Epitope retrieval was performed at 100°C for 20 min using the BOND Epitope Retrieval ER2 Solution (Leica Biosystems, AR9640), followed by endogenous peroxidase blocking for 5 min in 3 to 4% hydrogen peroxide. Multiplex immunostaining was performed in up to six successive cycles using the primary Ab-Opal fluorophore pairings; Ab dilutions and incubation periods are outlined in table S9.

Sections were counterstained with spectral DAPI (Akoya Biosciences, FP1490) and mounted with VECTASHIELD Vibrance Antifade Mounting Medium (Vector Laboratories, H-1700-10). Whole-slide scans and multispectral images were acquired at 40× magnification on a Vectra Polaris Automated Quantitative Pathology Imaging System (Akoya Biosciences). Batch analysis of multispectral images was performed with inForm Tissue Analysis software (version 2.4.8). Batch-analyzed multispectral images were stitched on the HALO image analysis platform (Indica Labs) to generate spectrally unmixed reconstructed images.

#### Analysis of mIF images

Reconstructed mIF images were analyzed using QuPath (version 0.5.0) (*102*). Cell segmentation was achieved using the built-in QuPath cell detection tool or StarDist (using the pretrained DSB 2018 model) (*103*) through the DAPI counterstain. Detected cells were classified as epithelial (i.e., malignant) or stromal or identified as artifactual (e.g., necrotic tissue) using supervised machine learning classifiers bespoke to each tumor specimen.

Random Trees object classifiers were trained on ≥10 manual annotations per compartment per tumor, guided by the DAPI and pCK channels, with training overseen by a consultant gastrointestinal histopathologist (A.E.). Stromal cells were further classified by immune marker positivity and normalized to the total number of cell detections in tissue sections.

To enable between-tumor comparisons of marker protein levels (e.g., pCK, cGAS, and STING), average marker intensity measurements for each cell were normalized to (divided by) its respective mean inferred autofluorescence value. Cancer and stromal compartment protein intensities were computed as the average of all the mean autofluorescence-normalized cellular intensities belonging to said compartment class.

#### cGAS^+^ MN detection

To identify cGAS^+^ MN (identified as small intense intracellular cGAS foci), tumor and stromal cell detections were merged into large stromal or tumoral cell regions. cGAS^+^ MN detection was carried out within these regions using the cell detection tool, focusing on the cGAS channel and applying area constraints of 0.25 to 7.5 μm^2^. The optimal cGAS intensity threshold for cGAS^+^ MN detection was determined for each tumor. cGAS^+^ MN detections in tumor cells were normalized to the total number of tumor cell detections, yielding a cGAS^+^ MN score (indicative of the degree of ongoing CIN) for each tumor.

#### CIN hotspot analysis

To map intratumoral regions with high cGAS^+^ MN densities (i.e., “CIN hotspots”), cGAS^+^ MN detections from cGAS–STING mIF panel–stained sections [manually annotated detections where available (*n* = 11 tumors]) or semiautomated detections where not (*n* = 24 tumors)] were first spatially aligned and projected onto adjacent (4 μm) immune panel–stained section images. Tumors with ≤10 cGAS^+^ MN detections were omitted from the analysis.

Kernel density estimation (KDE), using the “kde2d” function of the MASS package (version 7.3-60), was used to estimate the probability density function of cGAS^+^ MN across tumor images, and resulting kernel densities were quantized into 1% bins (ranging from 0 to 100%) to yield relative density distributions for each tumor.

To assess the enrichment of cell types within CIN hotspots, increasing density thresholds (in 1% increments, ranging from 25 to 95th percentiles) were used to define hotspot regions. Fold enrichment of cell types in CIN hotspots at each density threshold was calculated by comparing the relative proportion of a given cell type located inside versus outside the hotspot area. Tumors were excluded from the analysis at a given threshold if no cells of a given class were present within either the hotspot or the remainder region.

#### Response-specific CIN associations

Linear interaction models were used to examine patient response–specific associations between intratumoral immune cell infiltration metrics or compartment-specific cGAS–STING pathway component (cGAS and STING) protein expression and tumoral cGAS^+^ MN frequencies. Patient response status to NA Tx was inferred from histopathological response scores (TRG; also known as Mandard score). Patients with TRGs 1 and 2 were categorized as NA Tx responders, whereas patients with TRGs 4 and 5 were considered nonresponders (NR). The linear model was defined as follows

$${\text{Feature}}_{\left(X\right)}∼{\text{cGAS}}^{+}\;\text{MN}\times \text{Response status}$$

where response variables (Feature_(X)_) consisted of inferred intratumoral immune cell abundances or compartment-specific cGAS–STING pathway component protein expression, and the cGAS^+^ MN predictor variable consisted of the tumor-level cancer compartment–specific cGAS^+^ MN frequency.

Interaction term *P* values (cGAS^+^ MN × Response status) and the differences between slopes of cGAS^+^ MN with queried features between response classes (slope NR − slope R) were extracted to identify features showing preferential scaling with cGAS^+^ MN in one response class over another.

### Multiplex IMC

FFPE slides were dewaxed for 2 hours at 58°C, followed by incubations in xylene and successive concentrations of 100, 90, 70, and 50% ethanol. Samples were subjected to heat-mediated epitope retrieval in tris-EDTA (pH 9) at 98°C for 20 min. Tissues were then blocked with 3% BSA (A7906, Sigma-Aldrich) for 30 min at RT and stained overnight with the IMC Ab cocktail detailed in table S3 at 4°C. The following day, excess Ab was washed off with tris-buffered saline-Tween, nuclei were stained with DNA intercalator (201192A, Standard BioTools) at RT for 30 min, and slides were washed and left to dry, ready for laser ablation.

Image acquisition was performed using the Hyperion Fluidigm (Standard BioTools). Cell segmentation on resulting images was performed using the OPTIMAL pipeline (*104*). Clustering of cell types was performed using FCS Express software.

### *CXCL8* RNAscope

In situ detection of *CXCL8* mRNA transcripts in 4-μm FFPE human EAC tumor sections was carried out using the RNAscope assay (Advanced Cell Diagnostics) coupled to quantitative immunofluorescence on a Leica BOND Rx Autostainer using a 20-bp probe to the target region of *CXCL8* (2-1082, Advanced Cell Diagnostics). Tissue sections were pretreated with heat and protease before hybridization with oligonucleotide probes. Detection and amplification were performed with the RNAscope Leica Multiplex Fluorescent Assay (Advanced Cell Diagnostics). Image analysis and *CXCL8* mRNA quantification were performed using QuPath software (version 0.5.0).

Briefly, tissue compartment (malignant and stromal) masks were designed using supervised machine learning pixel classifiers (Random Trees) bespoke to each tumor specimen, trained on mIF-stained sections (pCK and DAPI) adjacent (4 μm) to RNAscope sections. Compartment masks were aligned onto *CXCL8* mRNA images, and images were thresholded to designate *CXCL8-*positive areas within tissue compartments. Resulting compartment-restricted *CXCL8*-positive areas were normalized to total tissue compartment areas as a percentage for each tumor.

### Transwell migration

#### Conditioned medium generation

To generate CM, EAC (OE33 and SK-GT-4) and CP-A cells were seeded at a density of 3.0 × 10^5^ or 5.0 × 10^5^ cells, respectively, in 100-mm cell culture dishes. After 24 hours, cell media were either replaced with fresh media or, where indicated, replaced with 1 μM MPS1i reversine (Cell Guidance Systems, SM85)– or DMSO-treated media for 24 h, followed by drug washout and replacement with fresh serum-reduced (5% FBS) media. Cell culture media were conditioned for 48 hours before collection and cleared by centrifugation at 1000*g* for 10 min at 4°C. For use in migration assays, conditioned media were further diluted with serum-free media (1:1 ratio) to a final concentration of 2.5% FBS. Migration controls included serum-free and unconditioned media (UM) as negative controls. UM were diluted with serum-free media, as described above. For positive controls, human recombinant IL-8 (Bio-Techne, 208-IL) or CCL2 (Proteintech, HZ-1334) were added to diluted UM.

#### CD14^+^ cell isolation

Human peripheral blood mononuclear cells were isolated from leukapheresis cones from healthy donors (NHS Blood and Transplant, UK) obtained under ethical approval reference REC 19LO1848 by Ficoll-Paque density centrifugation. CD14^+^ monocytic cells were isolated using human CD14 MicroBeads (Miltenyi Biotec, 130-050-201) and LS autoMACS Columns (Miltenyi Biotec, 130-042-401) as per the manufacturer’s protocol.

#### Transwell migration assay

CD14^+^ monocytes (5.0 × 10^5^ cells in 100 μl of UM) were added to the transwell inserts (6.5-mm transwells with 5.0-μm pores; Corning) and allowed to migrate for 24 hours toward CM or control media (600 μl) in the lower chambers. Where specified, CD14^+^ cells were incubated with CXCR1/2 inhibitor reparixin (50 μg/ml; Selleck Chemicals) for 30 min before seeding in transwell inserts in the presence of reparixin.

Migrated CD14^+^ cells in the lower chamber were imaged using a Celigo Image Cytometer (Nexcelom). At least two whole-well images were captured of each well, and a custom ImageJ script was used to determine cell counts for each image. Migration indexes were calculated by normalizing cell counts to the corresponding control well counts.

### TCGA analyses

#### Transcriptomic data access

Normalized [transcripts per million (TPM) and RNA-seq by expectation-maximization (RSEM)] and raw read RNA-seq counts for EAC tumors comprised in the TCGA (*105*) were accessed using the TCGAbiolinks R package (version 2.30.0) (*106*). Raw read counts were filtered, retaining genes with a total read count of ≥75 counts across all samples and nonzero expression in >25% of samples. Filtered genes were then DESeq2 normalized.

Raw read RNA-seq counts from TCGA STAD were accessed as described above and filtered to comprise only chromosomally unstable STAD tumors (STAD-CIN), as defined by previous molecular subtyping by the TCGA Research Network (*107*). Downstream filtering and normalization steps were performed as for EAC tumors.

#### Promoter methylation

Mean CpG-aggregated promoter methylation data for *CGAS* and *STING* from EAC tumor and adjacent esophageal tissue samples comprised in the TCGA were obtained through the SMART App web browser (available at www.bioinfo-zs.com/smartapp) (*108*).

#### Survival analyses

Gene signature scoring of patient tumors was performed on DESeq2-normalized RNA-seq counts with the GSVA R package (version 1.5.0) using the “single-sample GSEA” method (*109*). Gene sets used for GSVA scoring are listed in table S1.

Survival data were accessed using the TCGAbiolinks R package. Patient cohorts were dichotomized using the maximally selected rank statistic (“Max-Stat” method) with the maxstat package (version 0.7.25) (*110*). Univariate survival analyses were performed using the Kaplan-Meier (log-rank) test with the survival (version 3.5.7) R package (*111*).

### Linear models

#### CIN score agreement

Linear associations between orthogonal measures of CIN (aneuploidy, CIN^MN^, and CIN^70^ CIN signature scores) in EAC and STAD-CIN tumors were computed using linear regression models, accounting for tumor purity and leukocyte fraction as covariates. Tumor purity estimates [obtained from the ESTIMATE algorithm (*112*)], aneuploidy scores (*37*), and leukocyte fraction calls were obtained from source publications (*113*). CIN^70^ and CIN^MN^ signature scores were computed as described in the “Survival analyses” section. The models were defined as follows

$$\text{CIN}\;{\text{score}}_{\left(\text{CIN}70|\text{Aneuploidy score}\right)}∼\;{\text{CIN}}^{\text{MN}}\;+\;\text{Purity}\;+\;\text{Leukocyte fraction}$$

Resulting *P* values between orthogonal CIN scores were extracted to infer the significance of CIN score interrelation.

#### CIN-associated expression

Linear regression models to evaluate which genes are most strongly associated with CIN scores across EAC tumors were applied using DESeq2-normalized gene expression data. CIN scores were computed as described above. Linear models accounting for tumor sample leukocyte fraction and tumor purity were iterated over all detected genes in filtered gene expression matrices

$${\text{Expression}}_{\text{Gene}\;X}\;∼\;\text{CIN}\;\text{score}+\text{Purity}+\text{Leukocyte fraction}$$

where Expression represents DESeq2-normalized expression for a given gene, and the CIN score represent either CIN^70^ score, CIN^MN^ score, or aneuploidy score. CIN-associated pathway enrichment was computed through GSEA using *P* values indicative of the significance of association between CIN scores and gene expression, as well as the associated slope estimates of associations to compute gene rankings. Gene ranks for GSEA were computed as follows: –log_10_(*P* value) × sign(slope).

#### cGAS–STING–dependent CIN-associated expression

A linear interaction model was used to identify genes whose expression scaled with CIN^MN^ score in EAC tumors in a manner dependent on cGAS–STING expression. The CIN^MN^ signature and cGAS–STING expression scores were computed as described in the “Survival analyses” section. Tumor leukocyte fraction and tumor purity calls were included as covariates. Interaction models were defined as follows and iterated across all detected genes within the filtered DESeq2-normalized gene expression matrix

$${\text{Expression}}_{\text{Gene}\;X}∼\;{\text{CIN}}^{\text{MN}}\times \;\text{cGAS–STING}\;+\;\text{Purity}\;+\;\text{Leukocyte fraction}$$

The slopes and *P* values of associations between CIN^MN^ (the predictor variable) and gene expression (the response variable) were extracted from model outputs to determine which genes demonstrate significant (positive or negative) scaling with CIN^MN^ score across tumors. Slopes and *P* values of the interaction term were used to determine genes that exhibited a significantly different magnitude in association with CIN^MN^ score across different levels of cGAS–STING expression. Genes exhibiting significant positive associations (CIN^MN^ term *P* ≤ 0.05, positive slope) with CIN^MN^ and a significantly (interaction *P* ≤ 0.05) steeper slope across higher levels of cGAS–STING expression score were considered CIN enriched in a cGAS–STING–dependent manner. Conversely, genes exhibiting significantly negative associations with CIN^MN^ (CIN^MN^ term *P* ≤ 0.05, negative slope) and a significantly (interaction *P* ≤ 0.05) more negative slope across higher levels of cGAS–STING expression score were considered CIN depleted in a cGAS–STING–dependent manner. Functional enrichment of genes exhibiting a significant positive relationship with CIN^MN^ in a cGAS–STING–dependent manner was performed using overrepresentation analysis.

### CCLE analyses

#### Data access

Cell line aneuploidy scores and reads per kilobase of transcript per million reads mapped (RPKM)–normalized mRNA expression data were accessed through the DepMap portal (https://depmap.org/portal/) (*114*). RPKM expression data were log_2_(+1) normalized for visual comparison of relative expression levels between cell line types.

#### CIN score agreement

Linear associations between orthogonal CIN metrics (aneuploidy score and CIN^MN^ and CIN^70^ signature scores) among cell lines of the CCLE were computed using linear regression models, accounting for cell lineage as a covariate. CIN^70^ and CIN^MN^ signature scores were computed with the GSVA package (single-sample GSEA method) using RPKM-normalized expression data. The models were defined as follows

$$\text{CIN}\;{\text{score}}_{\left(\text{CIN}70|\text{Aneuploidy score}\right)}∼\;{\text{CIN}}^{\text{MN}}\;+\;\text{Lineage}$$

Resulting *P* values indicative of the significance of association between CIN scores were extracted.
