# Supplementary material for: Chromosomal instability shapes the tumor microenvironment of esophageal adenocarcinoma via a cGAS–chemokine–myeloid axis
Source: Sci Adv. 2026 Mar 11;12(11):eaeb1611. doi: 10.1126/sciadv.aeb1611 (PMC12978254; doi:10.1126/sciadv.aeb1611)
Supplement: Supplementary file 1 — Figs. S1 to S14 Legends for tables S1 to S9 [file sciadv.aeb1611_sm.pdf]

Science Advances
AAAS

Supplementary Materials for

Chromosomal instability shapes the tumor microenvironment of esophageal adenocarcinoma via a cGAS–chemokine–myeloid axis

Bruno Beernaert *et al.*

Corresponding author: Eileen E. Parkes, eileen.parkes@oncology.ox.ac.uk

Sci. Adv. **12**, eaeb1611 (2026)
DOI: 10.1126/sciadv.aeb1611

The PDF file includes:

Figs. S1 to S14
Legends for tables S1 to S9

Other Supplementary Material for this manuscript includes the following:

Tables S1 to S9

Supplementary Materials

Supplementary Figure 1. cGAS–STING expression across esophageal cancers and cell lines.

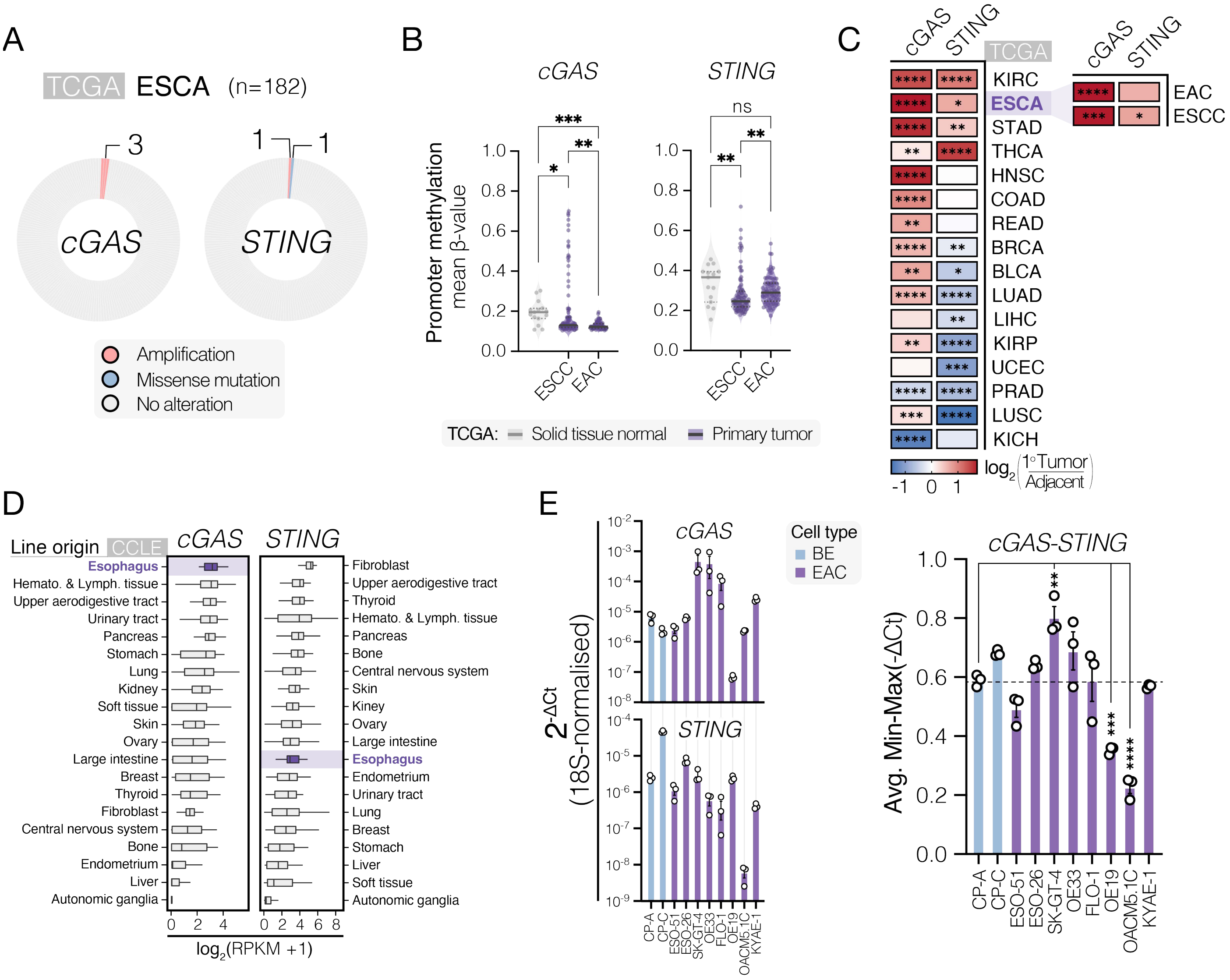

(**A**) Pie chart showing the mutations and copy-number alterations in the genes encoding cGAS and STING across 182 surveyed esophageal tumors (including EAC and ESCC tumors) comprised in the TCGA database.

(**B**) Violin plots of mean CpG-aggregated promoter methylation β-values levels in esophageal tumor samples (ESCA), including EAC (n=88) and ESCC (n=96), and putatively normal adjacent tissue (NAT) samples (i.e. 'solid tissue normal', n=15) from the TCGA for genes encoding cGAS and STING. Significance was determined by Kruskal-Wallis test with FDR-based correction.

(**C**) Heatmap of $\log_2$(fold-change) differences between primary tumor and putatively healthy adjacent tissue sample mRNA abundance (RSEM-normalized) of *cGAS* and *STING* for select solid tumor types comprised within the TCGA. Significance was determined by Mann-Whitney U test.

(**D**) Tukey box plots of $\log_2$-normalized *cGAS* (left panel) and *STING* (right panel) mRNA expression (RPKM+1) levels for Cancer Cell Line Encyclopedia (CCLE) cell lines grouped by cell line origin. Box plots are plotted in descending order based on median expression values. Box plots for esophagus-derived cancer cell lines are highlighted in purple.

(**E**) Quantitative reverse transcription (RT-qPCR) analysis of steady-state *cGAS* and *STING* mRNA expression levels across BE and EAC cell lines, normalized to the *18S* housekeeping gene. Average cGAS–STING expression represents the average of min-max-normalized -ΔCt values. Data from Barrett's esophagus (BE) cell lines are shown in light blue, data from EAC lines are shown in purple. Bars are shown as the mean ± SEM from n=3 independent experiments and were analyzed by one-way ANOVA with FDR-based correction. **** p ≤0.0001, *** p ≤0.001, ** p ≤0.01.

Supplementary Figure 2. Chromosomal instability features in esophageal adenocarcinoma cell lines.

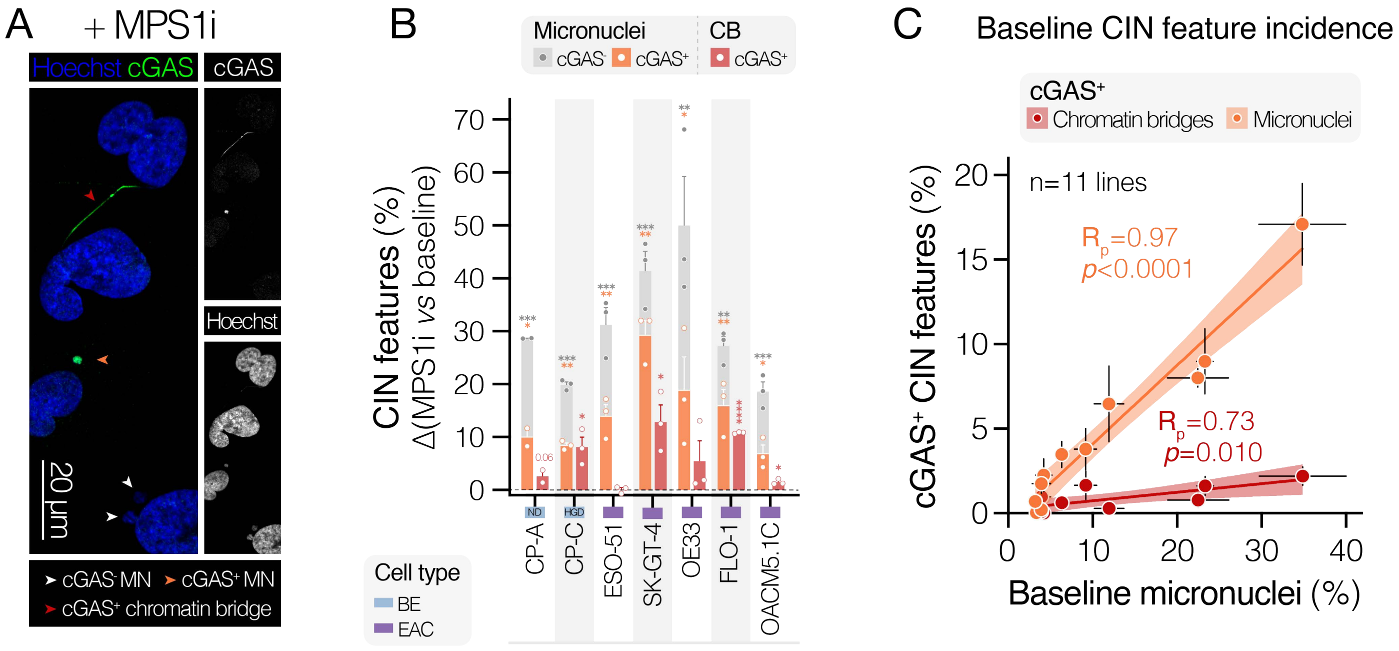

(**A**) Representative confocal microscopy image of SK-GT-4 cells following treatment with the MPS1 inhibitor reversine (0.5 µM) for 48h, comprising examples of scored CIN features, including cGAS- and cGAS+ micronuclei, as well as cGAS+ chromatin bridges. Cells were stained with anti-cGAS and Hoechst (DNA). The image represents a maximum-intensity projection of a Z-stack taken at 1 µm intervals. Scale bar corresponds to 20 µm.

(**B**) Difference in frequency (number of features / 100 cells) of scored CIN features between MPS1 inhibitor- (0.5 µM, 48h) and DMSO-treated cells. Barrett's esophagus (BE). Bars represent the mean ± SEM of n=2 (CP-A) or n=3 (all other lines) independent experiments, with ~≥100 cells counted per experiment. Data analyzed by two-sided unpaired t-test.

(**C**) Scatter plots of baseline cGAS+ chromosomal instability-associated feature (cGAS+ micronuclei and cGAS+ chromatin bridges) frequencies versus baseline micronuclei frequencies across BE and EAC cell lines. Dots represent the mean ± SEM of n=3 independent experiments, with ~≥100 cells counted per experiment. Shown are estimated simple linear regression lines, 95% confidence intervals, Pearson correlation coefficients (R_p) and p-values from Pearson correlation analyses. **** p ≤0.0001, *** p ≤0.001, ** p ≤0.01, * p ≤0.05.

Supplementary Figure 3. cGAS knockout and CIN-driven cGAS-dependent target validation in esophageal adenocarcinoma cells.

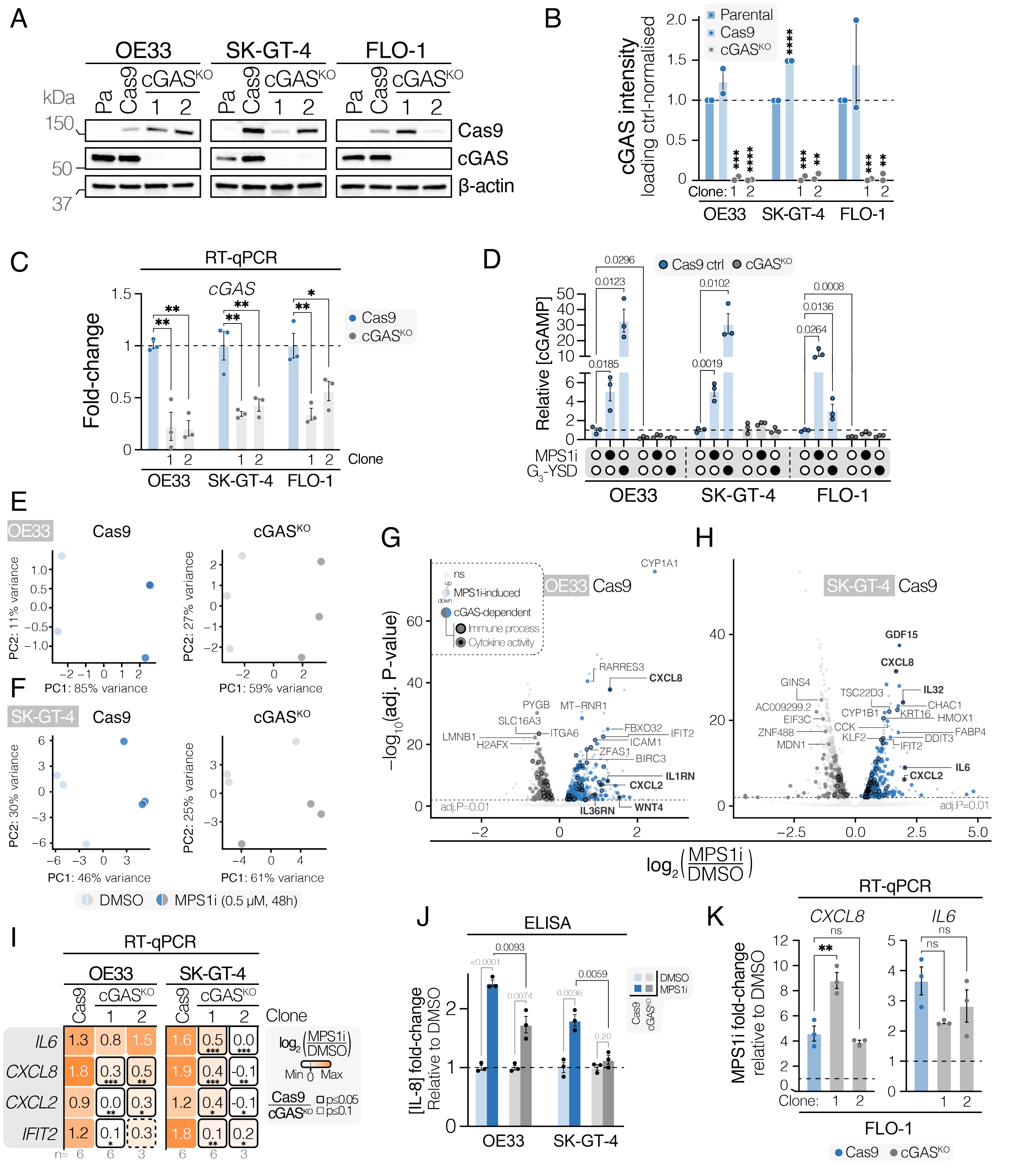

Supplementary Figure 3. cGAS knockout and CIN-driven cGAS-dependent target validation in esophageal adenocarcinoma cells.

(**A**) Representative immunoblot of parental, Cas9 control (empty-vector), and $cGAS^{KO}$ OE33, SK-GT-4 and FLO-1 cells. β-actin is used as a loading control. Data representative of n=2 experiments.

(**B**) Densitometry analysis of (**A**). Band intensities have been normalized to loading controls and parental cell cGAS protein intensity for each experiment. Bars are shown as the mean ± SEM (n=2). Analyzed by ANOVA with FDR correction for each cell line.

(**C**) RT-qPCR analysis of baseline *cGAS* mRNA expression levels across OE33, SK-GT-4 and FLO-1 Cas9 and $cGAS^{KO}$ clones. *CGAS* Ct values are normalized to the *18S* housekeeping gene using the ΔCT method and are expressed relative to normalized Cas9 control *cGAS* levels. Bars: mean ± SEM (n=3). Analyzed by ANOVA with FDR correction for each cell line.

(**D**) Relative extracellular concentrations of 2'3'-cGAMP (ELISA) across OE33, SK-GT-4 and FLO-1 $cGAS^{KO}$ (clone #1 for each) and Cas9 control clones. Cells have been pulse-treated with DMSO or 1 μM MPS1i (reversine) for 24h, followed by 48h release; or transfected with 2.5 μg/mL G_3-YSD 24h. Concentrations have been normalized to the DMSO-treated Cas9 control for each line. Bars: mean ± SEM (n=3). Analyzed by ANOVA with FDR correction for each cell line.

(**E, F**) Principal component analysis of transcriptomes of DMSO and MPS1i (0.5 μM, 48h)-treated Cas9 and $cGAS^{KO}$ clones of the (**E**) OE33 cell line and (**F**) SK-GT-4 line.

(**G, H**) Volcano plots of differential gene expression analysis in (**G**) OE33 and (**H**) SK-GT-4 cGAS-proficient Cas9 control cells between MPS1i-treated (0.5 μM, 48h) and control (DMSO)-treated cells. Genes highlighted in dark blue or dark grey are significantly more upregulated or downregulated (paired t-test $p \leq 0.05$) in Cas9 versus $cGAS^{KO}$ cells (i.e. 'cGAS-dependent') upon treatment. Genes related to 'Immune System Process' (GO:0002376) or with known 'Cytokine activity' (GO:0005125) are highlighted as indicated.

(**I**) Heatmap of RT-qPCR analysis of select immune targets in OE33 and SK-GT-4 Cas9 and $cGAS^{KO}$ clones treated for 48h with DMSO or 0.5 μM reversine (MPS1i). Color maps to $\log_2$-transformed fold-changes (FC) between MPS1i-treated cells and DMSO-treated cells. Solid borders indicate a significantly different $\log_2$(FC). Values inside heatmaps represent the mean $\log_2$(FC) across n=3 or n=6 experiments. Data analyzed by ANOVA with FDR correction, comparing $\log_2$(FC) induction in $cGAS^{KO}$ clones to the Cas9 control clone.

(**J**) IL-8 concentrations (ELISA) in conditioned media of cells pulse-treated with DMSO or 1 μM reversine (MPS1i) for 24h, followed by 48h release. Concentrations have been normalized to the respective DMSO-treatment per genotype. Grey and blue shades correspond to Cas9 and $cGAS^{KO}$ genotypes, respectively. Darker shades correspond to MPS1i-treatment. Bars: mean ± SEM (n=3). Data analyzed by ANOVA with FDR correction for each cell line.

(**K**) RT-qPCR analysis of *IL6* and *CXCL8* mRNA fold-induction upon MPS1i treatment in Cas9 and $cGAS^{KO}$ FLO-1 cells. Bars: mean ± SEM (n=3). Data were analyzed by ANOVA with FDR correction per gene. **** $p \leq 0.0001$; *** $p \leq 0.001$; ** $p \leq 0.01$; * $p \leq 0.05$; ns, not significant.

Supplementary Figure 4. Generation of an isogenic non-dysplastic Barrett's esophagus cell line model of variable chromosomal instability.

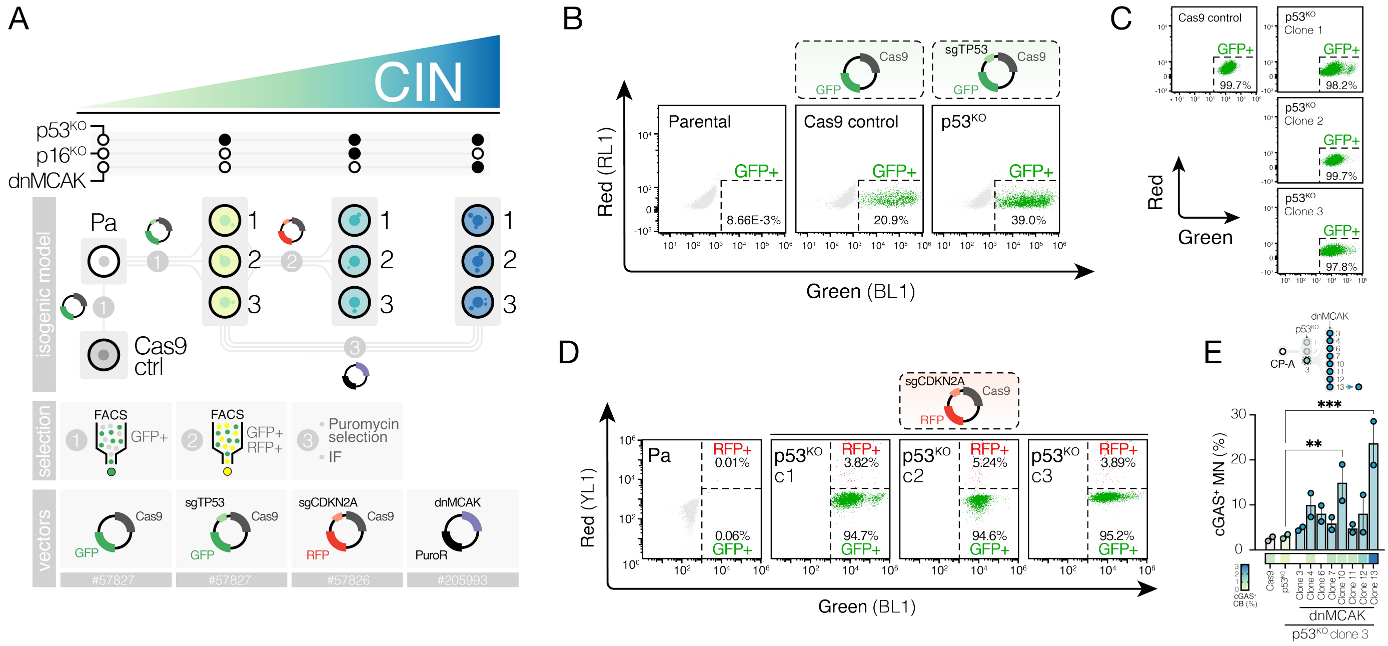

(**A**) Schematic of the experimental strategy used to generate an isogenic cell line model with varying levels of CIN, starting with an hTERT-immortalized, non-dysplastic BE-derived CP-A founder. Parental (Pa) CP-A cells were sequentially altered to disrupt intrinsic barriers to chromosomal instability, through CRISPR-Cas9-mediated targeting of *TP53* (encoding p53) and *CDKN2A* (encoding p16) and disruption of mitotic checkpoints through overexpression of a dominant-negative mutant form of the mitotic regulator MCAK (dnMCAK). A Cas9 control (Cas9 ctrl) clone, expressing pL-CRISPR.SFFV.eGFP, was generated to control for confounding effects associated with Cas9 overexpression. Clonal selections were performed through single-cell sorting, sorting GFP^{+} cells ($p53^{KO}$) and $GFP^{+}RFP^{+}$ cells ($p53p16^{DKO}s$), as well as through puromycin selection ($p53^{KO}dnMCAKs$, expressing the puromycin resistance [PuroR] gene) and limiting dilution, followed by immunofluorescence-based screening of micronucleation rates. Cloning was performed in triplicate, with three independent clones for every altered genotype (aside from Cas9 control cells) to avoid clone-specific confounding effects. Vector codes correspond to Addgene plasmid numers.

(**B**) Flow cytometric profiling of untransduced parental CP-A cells and CP-A cells transduced with CRISPR.SFFV.eGFP (Cas9 mixed population) and CRISPR.SFFV.eGFP.sgTP53 ($p53^{KO}$ mixed population) lentiviral vectors. Cells were gated on the GFP^{+} population for single-cell sorting.

(**C**) Flow cytometric profiling of expanded Cas9 and $p53^{KO}$ single-cell clones, gated on GFP^{+} as in (**a**), showing a universal uptake of CRISPR.SFFV.eGFP vectors among selected clones.

(**D**) Flow cytometric profiling of untransduced parental CP-A cells and $p53^{KO}$ clones transduced with CRISPR.SFFV.tRFP ($p53p16^{DKO}$ mixed population) lentiviral vectors. Cells were gated on the indicated $GFP^{+}RFP^{+}$ population for single-cell sorting.

(**E**) Example of IF-based screening of candidate $p53^{KO}$dnMCAK single-cell clones (derived from $p53^{KO}$ clone 3) for CIN^{high} clones, showing quantifications of $cGAS^{+}$ chromatin bridges (heatmap) and $cGAS^{+}$ micronuclei. Bars represent the mean of $cGAS^{+}$ MN frequencies ± SEM of n=2 technical replicates (two independent slides per clone, seeded in parallel), with ~≥100 cells counted per experiment. Data were analyzed by ANOVA with FDR correction, comparing all candidate clones to the Cas9 control clone. *** $p \leq 0.001$, ** $p \leq 0.01$.

Supplementary Figure 5. Validation of an isogenic non-dysplastic Barrett's esophagus model founder line and derived genotypes.

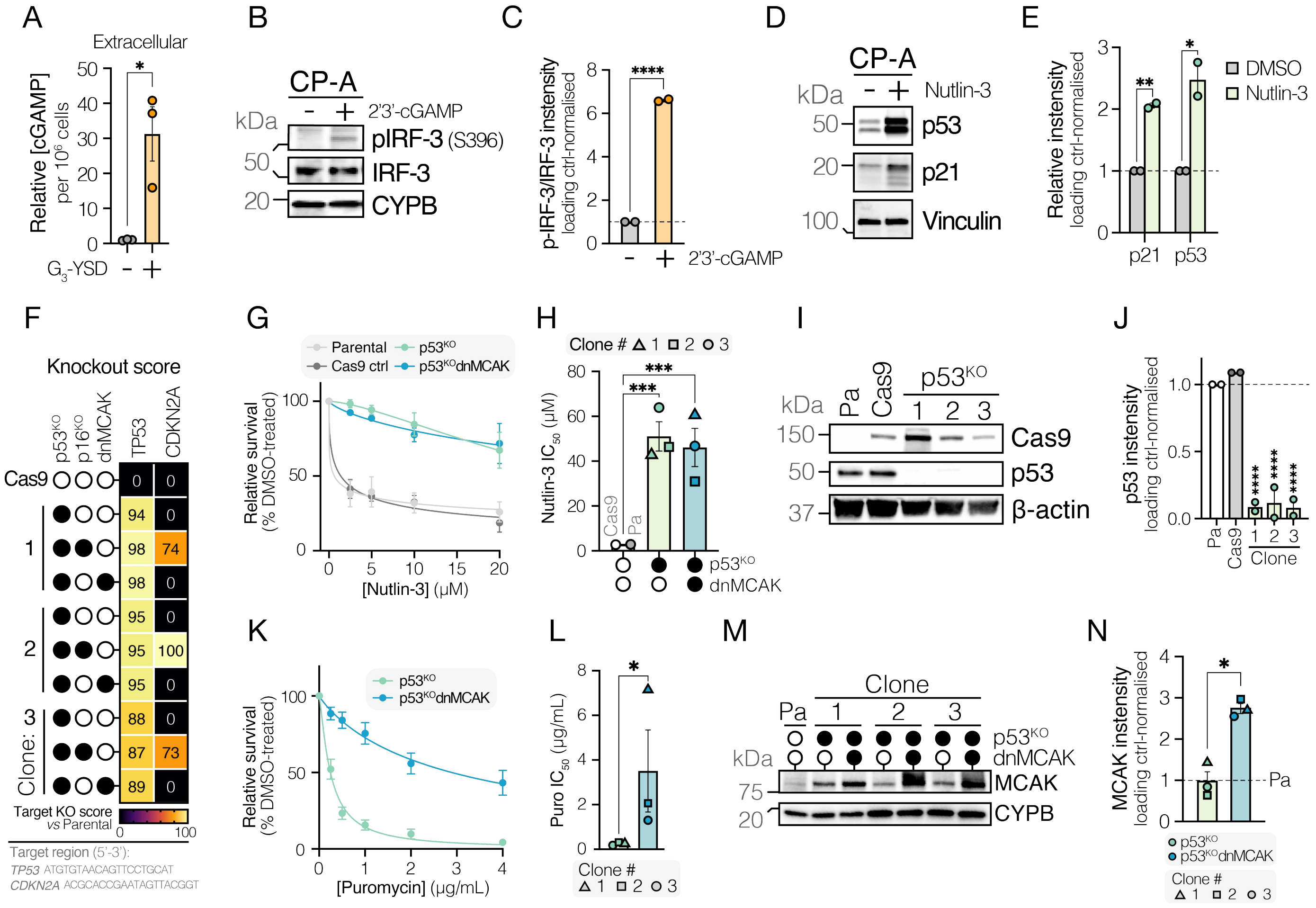

Supplementary Figure 5. Validation of an isogenic non-dysplastic Barrett's esophagus model founder line and derived genotypes.

(**A**) Relative extracellular concentrations of 2'3'-cGAMP (ELISA) in CP-A cells mock-transfected or transfected with 2.5 µg/mL G_3-YSD 24h prior to sample collection. Concentrations have been normalized to absolute cell counts ([cGAMP] / 10^6 cells) and are expressed relative to the mock. Bars are mean ± SEM (n=3). Data analyzed by two-sided unpaired t-test.

(**B**) Immunoblot of CP-A cells mock transfected or transfected with 10 µg/mL exogenous 2'3'-cGAMP. Representative of n=2 experiments. CYPB is used as a loading control

(**C**) Densitometric quantification of (**B**), showing the ratio between phosphorylated IRF3 (Ser396) and total IRF3 protein abundance. Band intensities have been normalized to CYPB abundance and are expressed relative to the mock. Bars: mean ± SEM (n=2). Data analyzed by two-sided unpaired t-test.

(**D**) Immunoblot of CP-A cells treated with diluent (DMSO) or 10 µm Nutlin-3 for 24h. Vinculin (VCL) is used as a loading control. Data are representative of n=2 experiments.

(**E**) Densitometric quantification of (**D**). Band intensities have been normalized to loading control abundance, expressed relative to normalized DMSO band intensities. Bars: mean ± SEM of n=2 experiments. Data analyzed by two-sided unpaired t-test.

(**F**) Heatmap of *TP53* and *CDKN2A* sgRNA target site knockout scores (Synthego ICE tool) for Cas9, $p53^{KO}$, $p53p16^{DKO}$ and $p53^{KO}$dnMCAK CP-A clones. Parental sequences have been used as a reference. Target sites are shown.

(**G**) Titration of Nutlin-3 in a 5-day viability (WST-8) assay for parental, Cas9 control, $p53^{KO}$ and $p53^{KO}$dnMCAK CP-A cells. Data are from n=3 experiments, with n=3 technical replicates per experiment. Data are represented as mean ± SEM. Genotype-level averages have been pooled for visualization purposes.

(**H**) Quantification of Nutlin-3 IC_{50} values from IC_{50} dose-response curves in (**G**). Bars are mean ± SEM of indicated genotypes. Datapoint shapes represent clone numbers. Significance was tested by one-way ANOVA with Tukey's HSD.

(**I**) Immunoblot of parental, Cas9 control and $p53^{KO}$ CP-A cells. Data are representative of n=2 experiments. β-actin is used as a loading control.

(**J**) Densitometry analysis of (**I**). Band intensities are normalized to the loading control and parental cell band intensities. Bars: mean ± SEM. Significance tested by one-way ANOVA with Tukey's HSD.

(**K**) Titration of puromycin in a 3-day viability (WST-8) assay for $p53^{KO}$ and $p53^{KO}$dnMCAK CP-A cells. Data are from n=3 experiments, with n=3 technical replicates per experiment. Data are represented as mean ± SEM. Genotype-level averages have been pooled for visualization purposes.

(**L**) Quantification of puromycin IC_{50} values from IC_{50} dose-response curves in (**K**). Bars: mean ± SEM of indicated genotypes. Datapoint shapes represent clone numbers. Significance tested by two-tailed ratio paired t-test.

(**M**) Immunoblot of parental, $p53^{KO}$ and $p53^{KO}$dnMCAK CP-A cells. Data are representative of n=3 experiments. CYPB is used as a loading control.

(**N**) Densitometry analysis of (**M**). Band intensities were normalized to the loading control and parental cell band intensities. Bars: mean ± SEM. Significance tested by two-tailed paired t-test. **** p ≤0.0001; *** p ≤0.001; ** p ≤0.01; * p ≤0.05.

Supplementary Figure 6. Validation of CIN-associated immune targets in an isogenic non-dysplastic Barrett's esophagus cell line model.

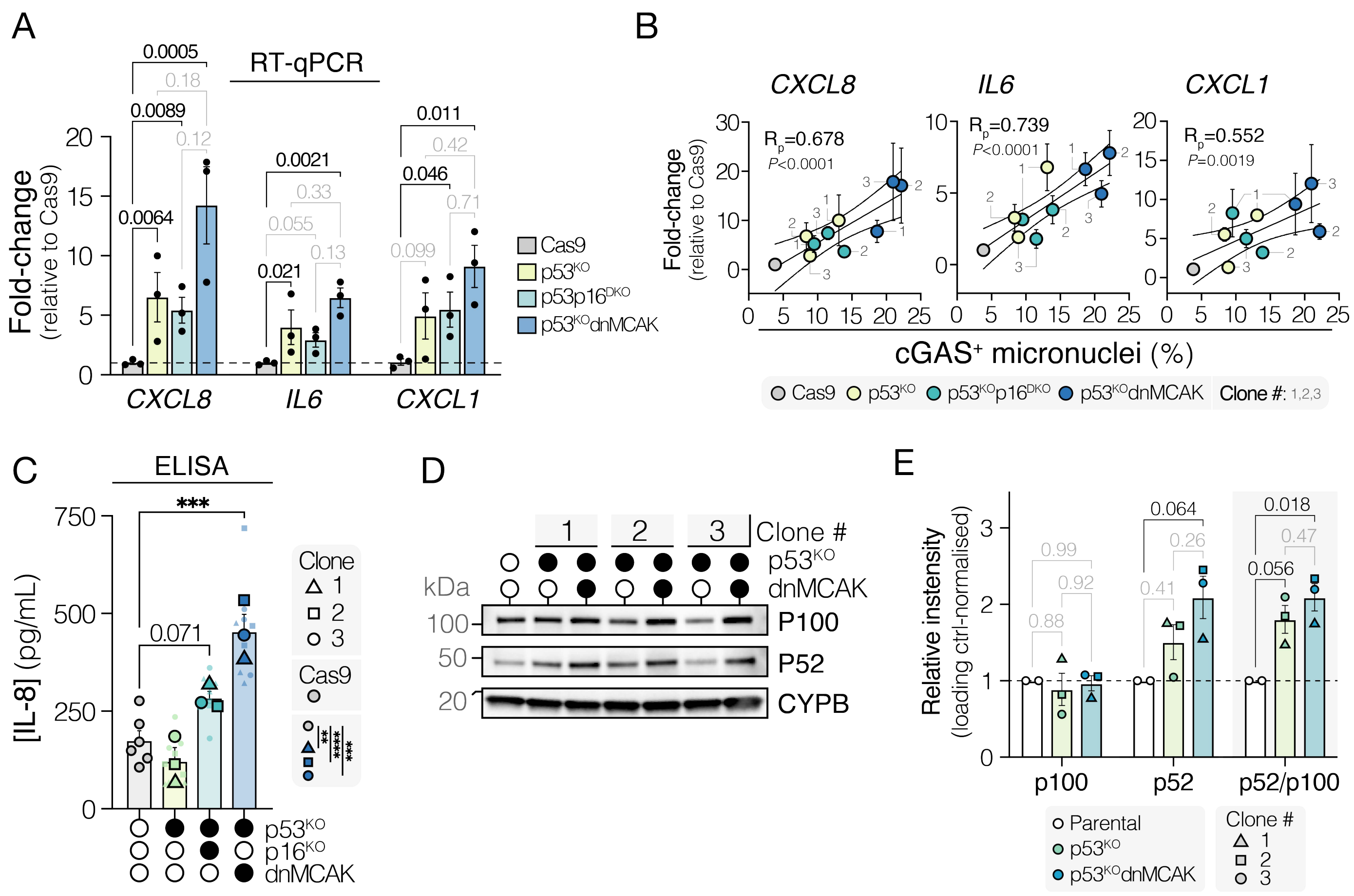

(**A**, **B**) Quantitative reverse transcription (RT-qPCR) analysis of baseline expression of the immune targets *IL6*, *CXCL8*, and *CXCL1* among CP-A cell model lines. Fold-changes were derived using the $2^{-\Delta\Delta Ct}$ method, normalizing expression values to *18S* expression and expression values in Cas9 control cells.

(**A**) Bars for $p53^{KO}$, $p53p16^{DKO}$, $p53^{KO}$dnMCAK genotypes represent the mean of n=3 independent clones ± SEM, where each point represents the mean of n=3 independent experiments for a given clone. Bars for Cas9 cells represent the mean ± SEM of n=3 independent experiments. Data were analyzed by ANOVA with FDR correction, comparing fold-changes in $p53^{KO}$, $p53p16^{DKO}$ and $p53^{KO}$dnMCAK genotypes to Cas9. Statistical analysis was performed on log_2-transfomed data.

(**B**) Linear relationship between baseline $cGAS^+$ MN burden for CP-A-derived cell lines and qPCR-derived relative expression levels of immune targets. Each point and its associated error bars represent the mean ± SEM of n=3 independent qPCR experiments (y-axis) and n=3 independent IF experiment (x-axis). Clone numbers are indicated. Pearson correlation analysis coefficients (R_p) and p-values are shown. Statistical analysis was performed on log_2-transfomed data.

(**C**) IL-8 concentrations (determined by ELISA) in conditioned media (conditioned for 48h) of CP-A-derived cell lines. Bars for $p53^{KO}$, $p53p16^{DKO}$, $p53^{KO}$dnMCAK genotypes represent the mean value across n=3 independent clones ± SEM, where each point represents the mean of n=3 independent experiments for a given clone. The bar for Cas9 cells represents the mean ± SEM of n=6 independent experiments. Datapoint shape indicates clone number for $p53^{KO}$, $p53p16^{DKO}$ and $p53^{KO}$dnMCAK clones. Data were analyzed by ANOVA with FDR correction, comparing genotype-level IL-8 concentrations in $p53^{KO}$, $p53p16^{DKO}$ and $p53^{KO}$dnMCAK genotypes to Cas9. Significant ANOVA p-values for individual clones (not pooled by genotype) are shown in the sidebar. *** p ≤0.001.

(**D**) Immunoblot of CP-A cell lines showing increased abundance of the non-canonical NF-κB pathway components p100 and p52. CYPB was used as a loading control. Data are representative of n=2 independent experiments.

(**E**) Densitometry analysis of (**D**). Band intensities are normalized to the loading control and parental cell band intensities. The ratio of p52 to p100 normalized protein abundance is shown. Bars for $p53^{KO}$ and $p53^{KO}$dnMCAK genotypes represent the mean of n=3 independent clones ± SEM, where each point represents the mean of n=2 independent experiments for a given clone. Bars for Cas9 cells represent the mean ± SEM of n=2 independent experiments. Data were analyzed by ANOVA with Tukey's HSD.

Supplementary Figure 7. Validation of a novel transcriptional signature of chronic ongoing chromosomal instability in esophageal cells.

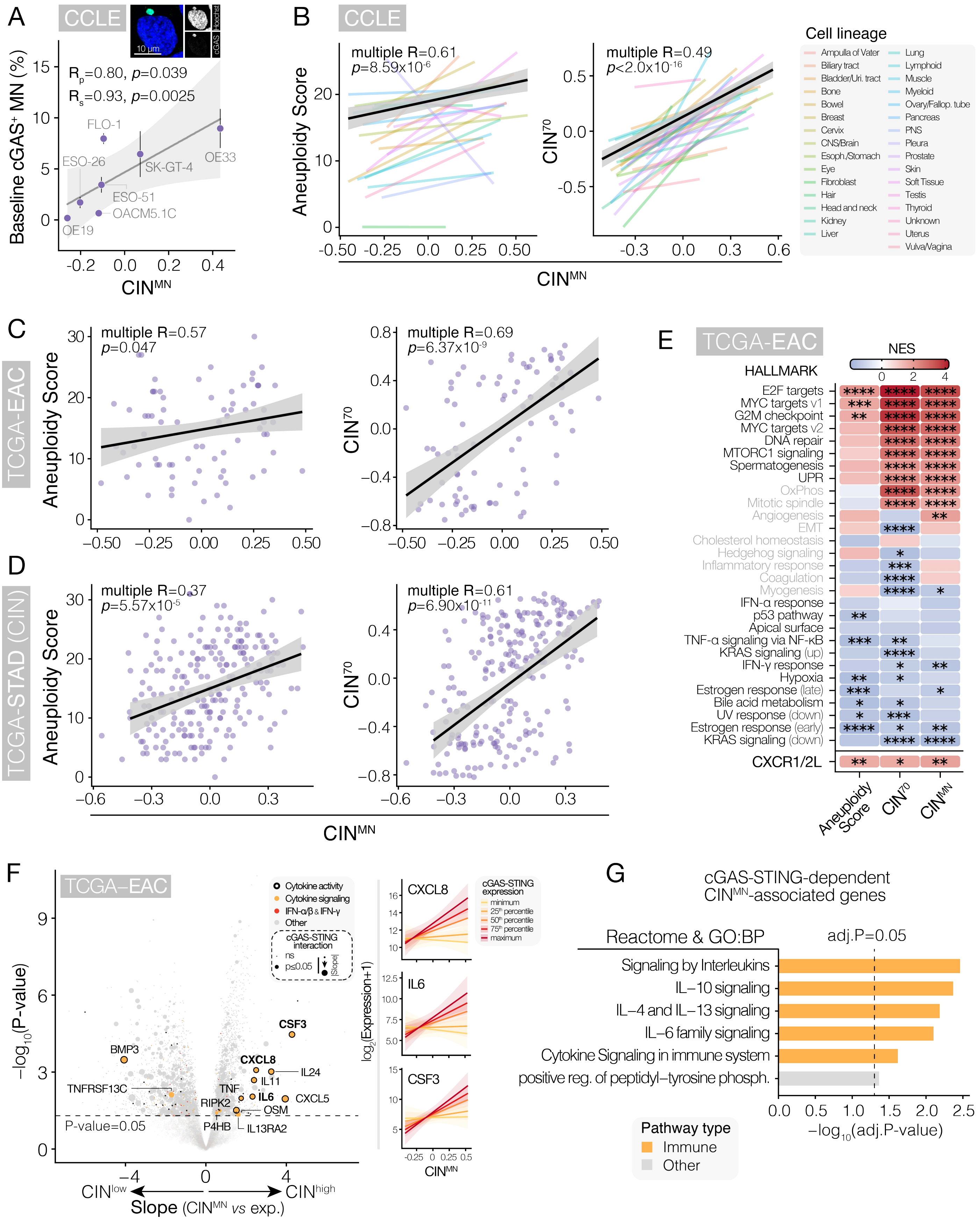

Supplementary Figure 7. Validation of a novel transcriptional signature of chronic ongoing chromosomal instability in esophageal cells.

(**A**) Scatter plot of microscopy-derived baseline $cGAS^+$ MN frequencies (exemplar image shown) versus CIN^{MN} scores (derived from CCLE RNA-sequencing data) for indicated EAC cell lines. The simple linear regression line, 95% confidence intervals, Pearson and Spearman correlation coefficients (R_p and R_s, respectively), and p-values are shown.

(**B**) Linear association between CIN^{MN} score and orthogonal metrics of CIN (CIN^{70} signature score and Aneuploidy score) across cell lines of the Cancer Cell Line Encyclopedia (CCLE). Colored lines represent simple linear regression lines in specific cell lineages. The significance of the association between CIN^{MN} and other CIN scores is derived using a linear model accounting for cell lineage as a covariate. The black regression line and grey 95% confidence intervals correspond to the marginal effect estimates of CIN^{MN} on CIN score over different cell lineages.

(**C**, **D**) Linear association between CIN^{MN} score and orthogonal metrics of CIN (CIN^{70} signature score and aneuploidy score) across (**C**) EAC tumors and (**D**) chromosomally unstable stomach adenocarcinoma (STAD-CIN) tumors comprised in The Cancer Genome Atlas (TCGA). The significance of the association between CIN^{MN} and other CIN scores is derived using a linear model accounting for tumor purity and leukocyte fraction as covariates. The black regression line and grey 95% confidence intervals correspond to the marginal effect estimates of CIN^{MN} on CIN score over covariates.

(**E**) Heatmap of gene set enrichment analysis (GSEA) outputs, showing MSigDB Hallmark gene sets that are most strongly commonly enriched with orthogonal CIN metrics (Aneuploidy score, CIN^{70}, CIN^{MN}) in EAC tumors from the TCGA. GSEA of a CXCR1/2 ligand (CXCR1/2L; *CXCL1–3*, *CXCL5–8*) signature shows a positive enrichment across all three CIN metrics. **** $p \leq 0.0001$; *** $p \leq 0.001$; ** $p \leq 0.01$; * $p \leq 0.05$.

(**F**) Volcano plot showing outputs of a linear model looking at the association between CIN^{MN} score and gene expression that are conditional on cGAS–STING expression (whilst accounting for leukocyte fraction and tumor purity as confounders) in EAC tumor from the TCGA. Slopes and p-values of genes indicate the strength of association with CIN^{MN} across EAC tumors. Genes that have an additional dependence (i.e. a statistically significant interaction; interaction term $p \leq 0.05$) on cGAS–STING expression are highlighted through size. Genes annotated with the 'Cytokine signaling' gene ontology term (GO:0019221) are highlighted in orange. Genes with known IFN-α/β (R-HSA-909733) or IFN-γ (R-HSA-877300) pathway involvement are highlighted in red. Genes with reported cytokine activity (GO:0005125) are marked by a border. The right panel includes examples of linear model predictions of the relationship between select genes (*CXCL8*, *IL6*, *CSF3*) across multiple levels of tumoral cGAS–STING expression.

(**G**) Overrepresentation analysis (using Reactome and Gene Ontology: Biological Process pathway sets) of cGAS-dependent CIN-associated genes (i.e. genes that scale significantly more strongly in high cGAS–STING expression settings) showing a statistical overrepresentation of inflammatory pathways.

Supplementary Figure 8. Detection of cGAS^{+} micronuclei as a measure of ongoing chromosomal instability in human EAC tumors.

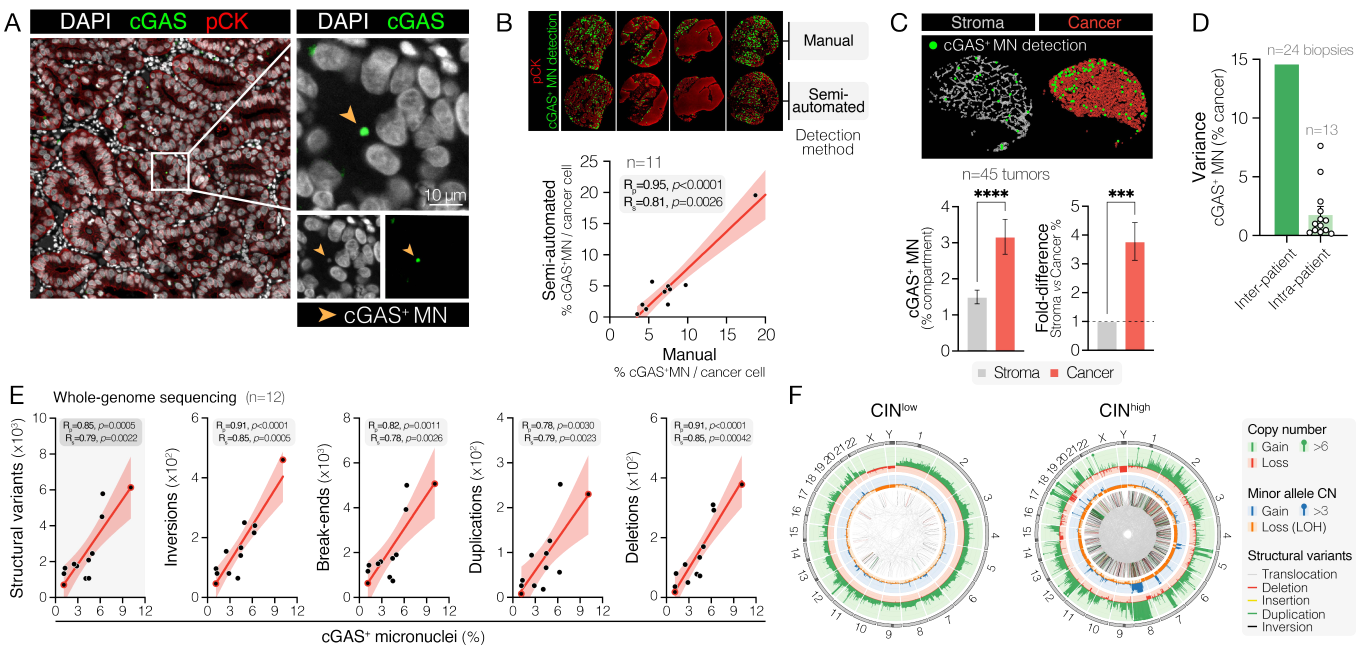

(**A**) Representative high-resolution image of a human EAC tumor biopsy specimen stained with DAPI (DNA), anti-cGAS and anti-pan-cytokeratin (pCK) antibody showing selective localization of cGAS at micronuclei. Scale bar corresponds to 10 µM.

(**B**) *Upper panel:* Examples of manual and semi-automated tumoral cGAS^{+} MN detections.

Lower panel: Scatter plot of manual cGAS^{+} MN quantifications ('ground truth') versus quantifications obtained using a semi-automated detection approach across n=11 EAC tumor specimens, showing high concordance between methods. Detections were limited to the cancer cell compartment and were normalized to the total number of detected cancer cells to obtain cancer compartment-specific cGAS^{+} MN frequencies. The simple linear regression line, 95% confidence intervals and Pearson and Spearman correlation coefficients (R_p and R_s, respectively) and p-values are shown.

(**C**) *Upper panel:* Example of semi-automated detections in the stromal versus cancer cell compartment of a primary EAC tumor.

Left lower panel: Bar plots of semi-automated cGAS^{+} MN detections in cancer versus stromal compartments across n=45 patient tumors, showing a higher preponderance of micronuclei in malignant compartments.

Right lower panel: Bar plots showing the fold-difference in cGAS^{+} MN detection frequencies in cancer compartments relative to corresponding stromal compartments.

Bars represent the mean ± SEM for n=45 human EAC tumors. Data were analyzed by Wilcoxon matched-pairs signed-rank test, pairing malignant and stromal values from the same tumors. **** $p \leq 0.0001$; *** $p \leq 0.001$.

(**D**) Bar plot comparing inter- and intra-patient variances in tumoral cGAS^{+} MN frequencies, showing lower variance among samples derived from the same patient (e.g. patients with matched pre-treatment biopsies and post-treatment resections) compared to pre-treatment samples from different patients. Bars represent the mean ± SEM.

(**E**) Scatter plots of the observed tumoral cGAS^{+} MN frequency versus whole-genome sequenced (WGS) -derived structural variant (SV) loads across n=12 primary EAC tumor specimens. The simple linear regression line, 95% confidence intervals and Pearson and Spearman correlation coefficients (R_p and R_s, respectively) and p-values are shown. Datapoints used as exemplars in (**f**) are highlighted with a red border.

(**F**) Circos plots showing copy number alterations (CNAs) and SVs for representative EAC tumors exhibiting the highest and lowest measured cGAS^{+} MN frequencies across all sequenced tumors (CIN^{high} and CIN^{low}, respectively). The outermost circle shows chromosomes, with darker shading representing gaps in the reference human genome (e.g. centromeres, heterochromatin and missing short arms). The second circle shows tumor purity-adjusted copy number (CN) changes, with gains shown in green and losses shown in red. Absolute copy numbers > 6 are shown with a green dot. The third shell represents minor allele CNs, with gains and losses shown in blue and orange, respectively. Minor allele CN > 3 are highlighted with a blue dot. The innermost circle displays intra- and inter-chromosomal SVs. SV categories are highlighted by color, as indicated.

Supplementary Figure 9. Single-nucleus RNA sequencing of human EAC tumors.

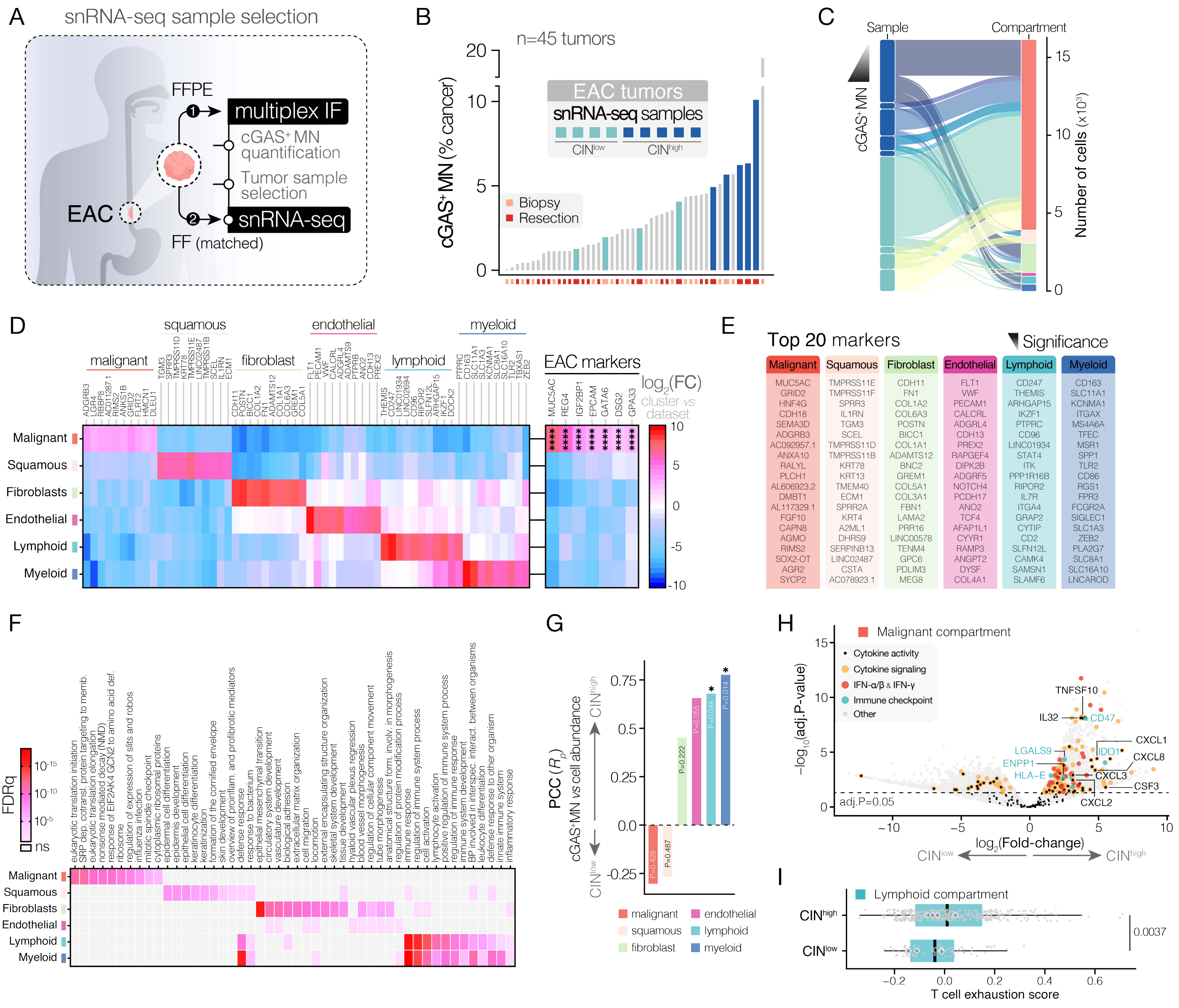

Supplementary Figure 9. Single-nucleus RNA sequencing of human EAC tumors.

(**A**) Experimental strategy used to shortlist samples for single-nucleus RNA-sequencing (snRNA-seq). Matched fresh frozen (FF) and formalin-fixed paraffin-embedded (FFPE) tissue specimens were obtained for tumors. FFPE tissues were stained with a multiplex immunofluorescence (mIF) panel using antibodies targeting cGAS and pan-cytokeratin (pCK), as well as DAPI (DNA) to evaluate the range of CIN across n=45 patient tumors. Tumors for snRNA-seq were shortlisted to span the observed spectrum of CIN across all queried tumors. SnRNA-seq was performed on matched FF tissue.

(**B**) Multiplex immunofluorescence (mIF)-derived $cGAS^+$ MN frequencies across n=45 patient tumors, including treatment-naïve staging laparoscopy biopsies and surgical resection specimens.

(**C**) Sankey plot showing the contribution (number of cells) of each sample to identified cell compartments. Samples are ranked in descending order according to their mIF-inferred $cGAS^+$ MN burden.

(**D**) Heatmap showing the top 10 most significant highly expressed markers associated with each of the identified cell clusters. Color maps to the log_2-transformed fold-change of a given marker in a cell cluster versus all other cell clusters. Select well-established EAC markers are indicated, showing high specific expression in the malignant compartment.

(**E**) The top 20 most significantly enriched markers in each cluster, ranked from top to bottom by significance of enrichment. Unlike (**C**), shown markers are not limited to genes with abundant expression (i.e. minimum 1 count / cell).

(**F**) Heatmap of the top 10 most significantly positively enriched pathways in each cluster, showing cluster specific pathway activities aligned with cluster identities. Pathway enrichment was performed using gene set enrichment analysis (GSEA) on 'FindAllMarkers' differential expression analysis outputs for each cluster. Color maps to the $-log_{10}$-transformed significance of GSEA enrichment (FDRq). Cluster-enriched pathways are ranked from left to right within each cluster by significance of enrichment.

(**G**) Bar plots of the Pearson correlation coefficients (R_p) of the relative abundance of each cell compartment (the percentage of cells of a given identity relative to all cell detection within a sample) versus the tumoral $cGAS^+$ MN burden across samples. P-values of Pearson correlation analyses are shown.

(**H**) Volcano plot showing differential gene expression between malignant cells of CIN^{high} and CIN^{low} snRNA-seq samples. Genes annotated with the 'Cytokine signaling' gene ontology term (GO:0019221) are highlighted in orange. Genes with known IFN-α/β or IFN-γ (R-HSA-909733 or R-HSA-877300, respectively) pathway involvement are highlighted in red. Genes with reported cytokine activity (GO:0005125) are marked by a black central dot. Common tumor-expressed checkpoints (see **Table S1**) are highlighted in teal. The dashed line indicates a Benjamini-Hochberg-adjusted p-value of 0.05.

(**I**) Box plots of T cell exhaustion scores in lymphoid cells from CIN^{high} and CIN^{low} snRNA-seq samples. T cell exhaustion scores were computed using the 'AddModuleScore' function of the Seurat package using a literature-curated T cell exhaustion signature (**Table S1**). Boxes represent the median ± interquartile range and whiskers were plotted using Tukey's method. Mean scores for each sample are plotted. Significance was determined by Mann-Whitney U test. **** $p \leq 0.0001$; * $p \leq 0.05$.

Supplementary Figure 10. Imaging mass cytometry-based profiling of the chromosomally unstable EAC tumor immune landscape.

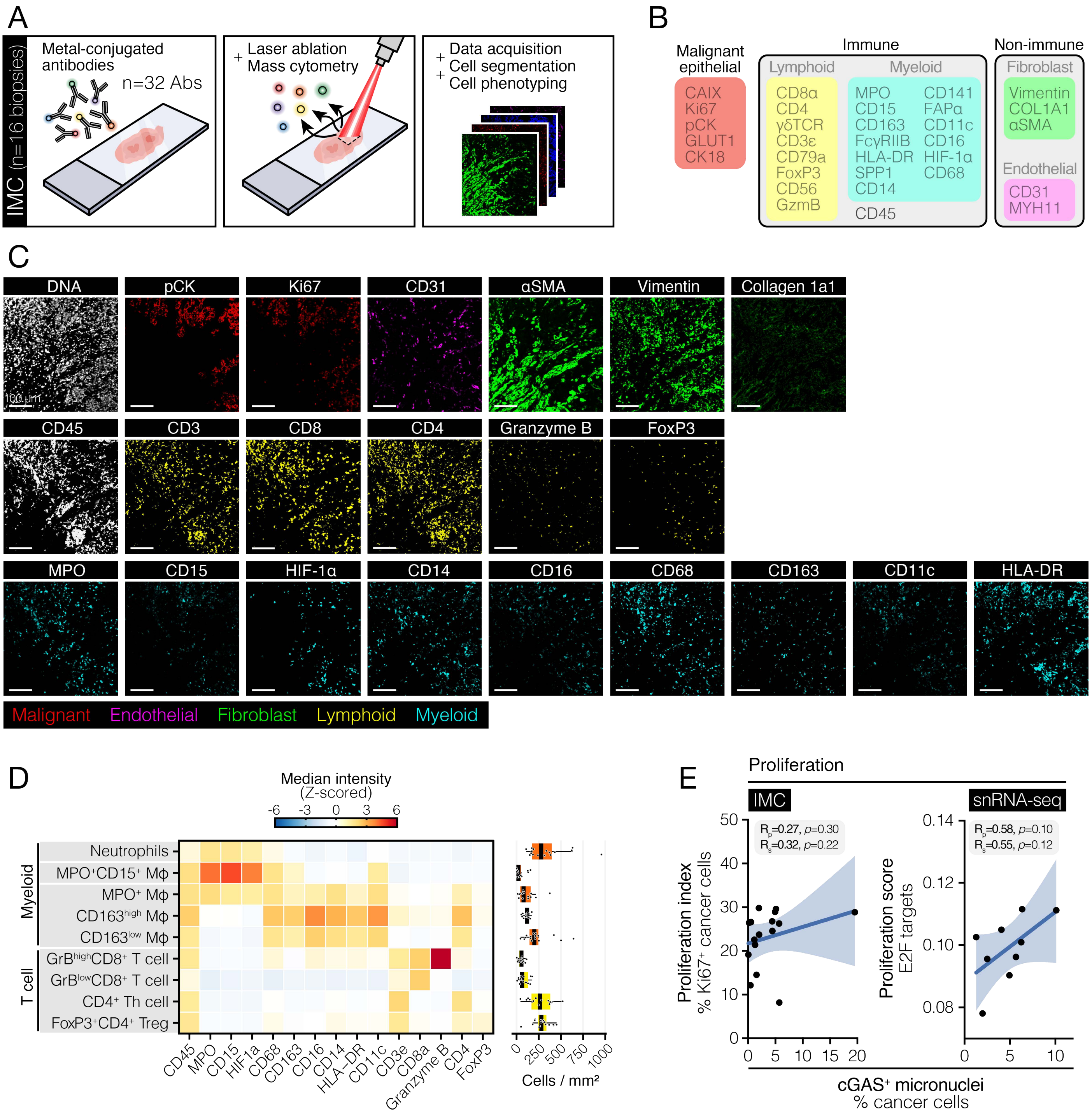

(**A**) Multiplexed imaging mass cytometry (IMC) workflow applied across n=16 pre-treatment human EAC tumors.

(**B**) Schematic of the IMC antibody panels used for human EAC tumor microenvironment phenotyping, showing the targets used to differentiate malignant epithelial, immune (lymphoid and myeloid) and non-immune stromal (fibroblasts and endothelial cells) cells.

(**C**) Representative images of various malignant, immune and non-immune stromal cell IMC markers in a pre-treatment human EAC tumor region. Scale bars correspond to 100 µM.

(**D**) *Left panel:* Heatmap of median Z-scored (on a per sample basis) intensities of markers from the immune panel across identified immune cell subtypes.

Right panel: Box plots of cell densities of identified immune cell subtypes across n=16 primary EAC biopsies. Boxes represent the median ± interquartile range, whiskers were plotted using Tukey's method.

(**E**) Scatter plots of tumoral cGAS^{+} MN frequencies versus proliferation scores across human EAC tumors. *Left panel:* Proliferation index across IMC tumors was determined as the proportion of Ki67^{+} tumor cells.

Right panel: Proliferation score was computed through the 'AddModuleScore' function of the Seurat package using a proliferation-associated gene signature (E2F targets, MSigDB Hallmark; **Table S1**).

Simple linear regression lines, 95% confidence intervals, Pearson and Spearman correlation coefficients (R_p and R_s, respectively), and associated p-values are shown.

Supplementary Figure 11. Multiplex immunofluorescence-based profiling of the chromosomally unstable EAC tumor immune landscape.

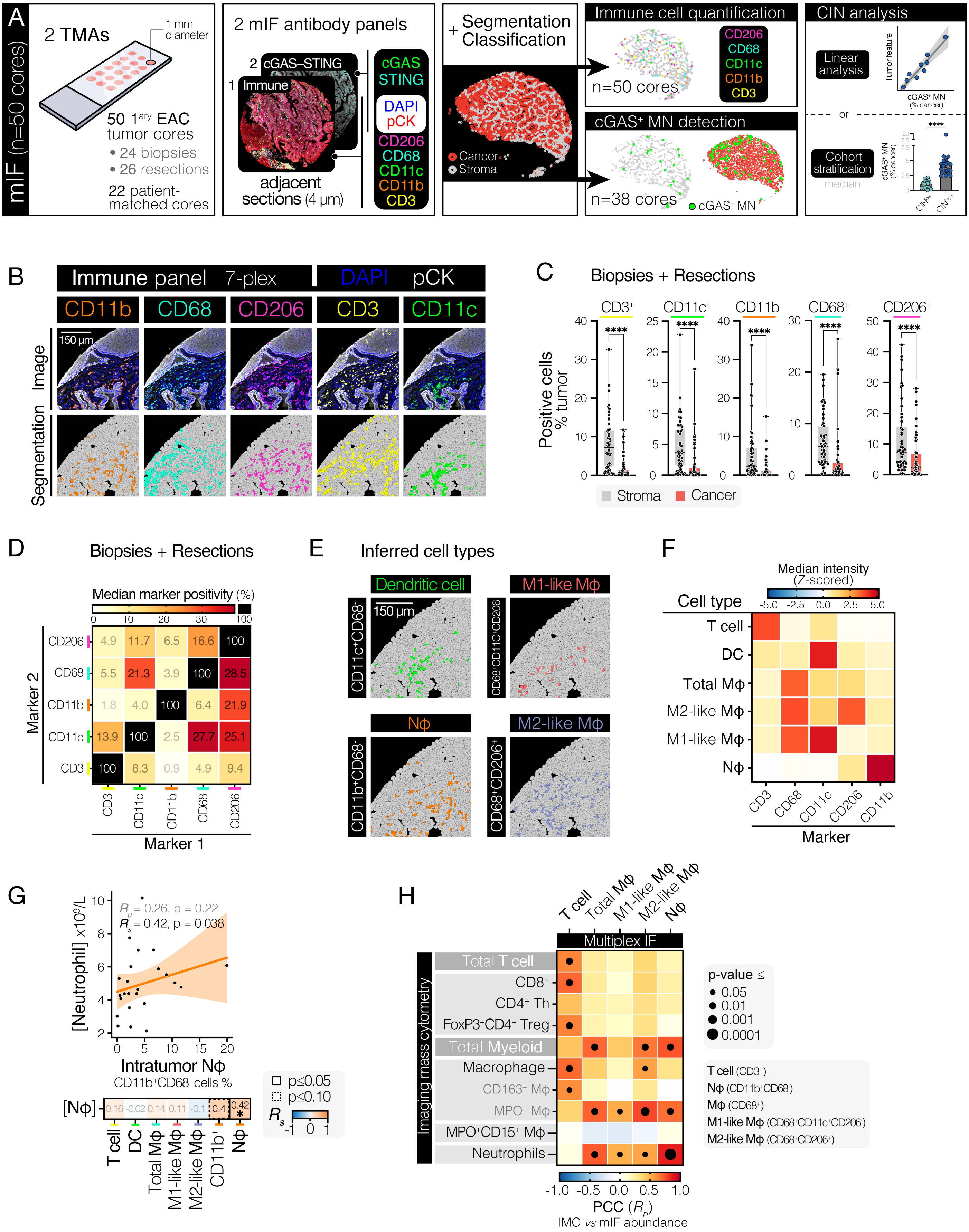

Supplementary Figure 11. Multiplex immunofluorescence-based profiling of the chromosomally unstable EAC tumor immune landscape.

(**A**) Multiplex immunofluorescence workflow. For CIN^{high} (n=19 EAC tumors) versus CIN^{low} (n=19) median stratification of the tissue microarray (TMA) sample cohort, bars represent the mean ± SEM. Significance was determined via Mann-Whitney U test.

(**B**) *Image*: Representative high-resolution image of a human EAC tumor biopsy specimen stained with DAPI (DNA), as well as anti-pan-cytokeratin (pCK), anti-CD11b, anti-CD68, anti-CD206, anti-CD3 and anti-CD11c antibodies (Abs; 7-plex staining), showing broadly stromal staining patterns for immune cell markers.

Segmentation: Representative cell segmentations showing immune marker-positive cell detections after setting appropriate intensity thresholds. Scale bar corresponds to 150 μM.

(**C**) Box plots showing the number of stromal compartment versus cancer compartment immune marker-positive cell detections (as a percentage of all cell detections in a tumor) across all analyzed biopsy and resection specimens, showing a predominance of immune marker-positive cells in stromal compartments. Boxes represent the median ± interquartile range. Box plot whiskers range from minimum to maximum. Data were analyzed by Wilcoxon matched-pairs signed-rank test, pairing malignant and stromal detections from the same tumors.

(**D**) Coincidence heatmap of the degree of marker positivity co-occurrence across all detected cells in human EAC biopsy and resection specimens, showing a pronounced co-occurrence (e.g. CD68 and CD206) or mutual exclusivity (e.g. CD11b and CD3) between some immune markers. Color maps to the median marker positivity across all tumors.

(**E**) Representative cell segmentations in an EAC biopsy tumor showing multi-marker cell detections indicative of inferred cell types. Scale bar corresponds to 150 μM.

(**F**) Heatmap of median Z-scored (on a per sample basis) intensities of markers from the mIF immune panel across identified immune cell subtypes.

(**G**) Scatter plot of the abundance of infiltrating neutrophils (Nϕ) in pre-treatment tumors versus concurrently collected blood-circulating neutrophil counts [Nϕ]. The heatmap shows spearman correlations coefficients (R_s) of associations between circulating neutrophil counts and all detected intratumoral cell types. Heatmap color maps to R_s. R_s values of correlations are shown inside the boxes. Pairwise correlations with a $p \leq 0.05$ are highlighted with a solid black border, whereas correlations with a $p \leq 0.10$ are highlighted with a dashed black border. The linear regression line, 95% confidence interval, R_s and Spearman correlation p-value of the association between Nϕ abundance and circulating [Nϕ] are shown on the scatter plot.

(**H**) Correlation heatmap showing high inter-correlations between immune cell type abundances inferred through imaging mass cytometry (IMC)-based immunophenotyping and through mIF across n=16 human EAC tumors analyzed using both methods. Color maps to the Pearson correlation coefficient (R_p). Significant associations in the abundance between cell types inferred through different methods are highlighted with a central black dot. Dot size maps to the magnitude of significance, as indicated. **** $p \leq 0.0001$; * $p \leq 0.05$.

Supplementary Figure 12. Tumor cell-intrinsic and stromal *CXCL8* mRNA expression in human EAC tumors.

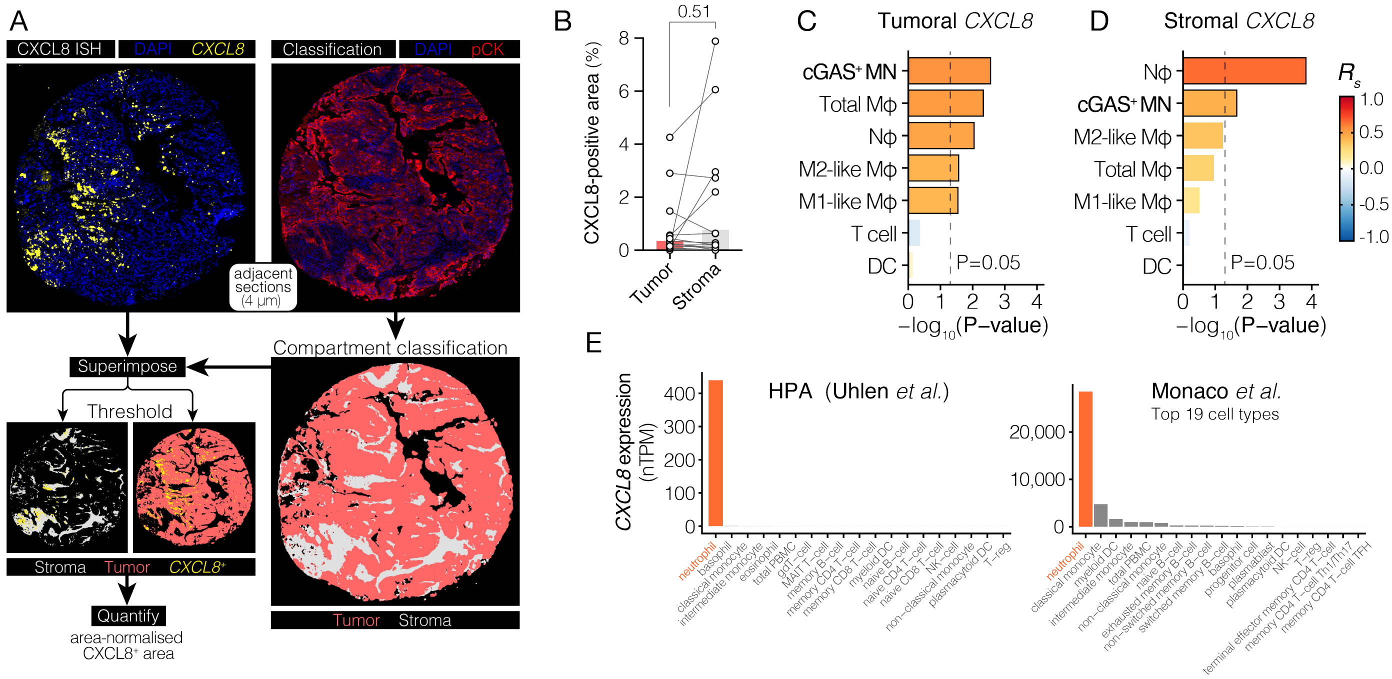

(**A**) Workflow to quantify malignant and stromal compartment-specific *in situ CXCL8* expression in human EAC tumors from *CXCL8* RNAscope images. Example tumor images correspond to exemplars used in **Fig. 5I** (CIN^{high}, top panels).

(**B**) Tumoral (cancer cell-intrinsic) versus stromal *CXCL8* expression across n=29 EAC tumors. Bars represent the mean and lines between datapoints link tumor and stromal values for a given sample. Data were analyzed by Wilcoxon matched-pairs signed-rank test, pairing malignant and stromal *CXCL8* expression from the same tumors.

(**C**, **D**) Bar plots of Spearman correlations of the association between (**C**) Tumoral or (**D**) stromal *CXCL8* expression and CIN (tumoral $cGAS^{+}$ MN frequency) or intratumoral immune cell abundance. Color maps to Spearman correlation coefficient (Rs). The x-axis corresponds to the –log_{10}-transformed Spearman correlation p-value. The dashed line corresponds to a p-value of 0.05.

(**E**) *CXCL8* mRNA expression level across peripheral blood mononuclear cell (PBMC)-derived immune cell subtypes in the Human Protein Atlas(*40*) and the Monaco *et al.*(*41*) datasets.

Supplementary Figure 13. Chromosomal instability-driven cGAS–STING as a monocyte-attracting cue.

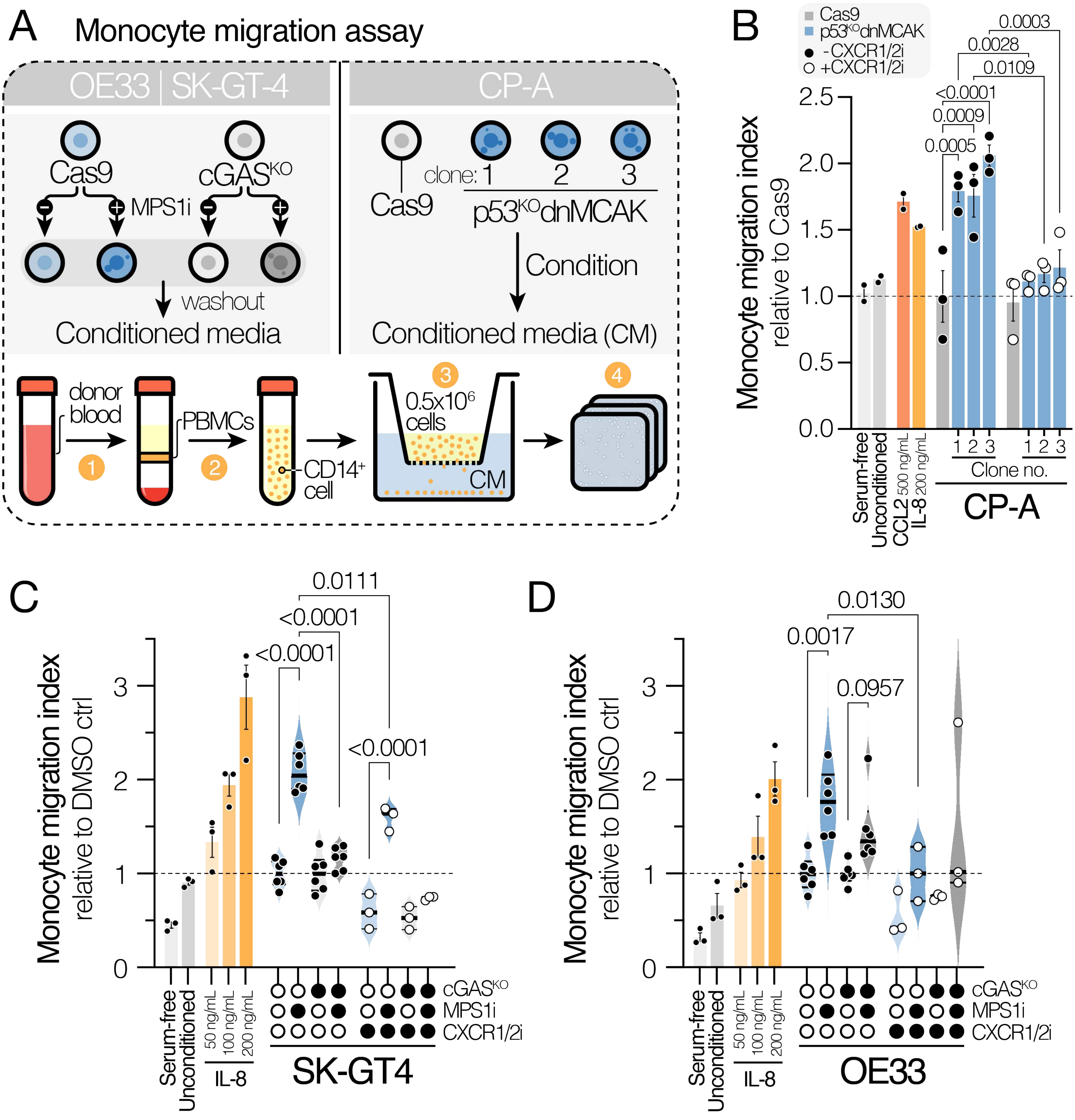

(**A**) Schematic illustrating the experimental strategy for monocyte migration assays. For EAC cells, Cas9 and cGAS^{KO} cells were exposed to 1 µM MPS1i (reversine) for 24h, followed by extensive wash-out to avoid drug carry-over. Cells were then allowed to condition media for 48h prior to conditioned media (CM) collection. For CP-A cells, cells were covered in fresh untreated media and left to condition media for 48h. CD14^{+} monocytes were positively selected from healthy donor blood-derived peripheral blood mononuclear cells (PBMCs). Migration assays were performed using transwell migration chambers, allowing monocytes to migrate towards CM for 24h before whole-well imaging based quantification.

(**B**) Transwell assays were performed as described in (**A**). Data were normalized to the untreated Cas9 control. Data of n=3 independent experiments are shown. Significance was tested by one-way ANOVA with FDR-correction.

(**C**, **D**) Transwell assays were performed as described in (**A**), using (**C**) SK-GT-4 cell conditioned media (CM) or (**D**) OE33 CM. Migration indexes were normalized to respective DMSO controls. Data of n=6 independent experiments are shown for experimental samples and n=3 for migration control samples. Monocytes were derived from n=2 independent healthy donors. Significance was tested by one-way ANOVA with FDR-correction.

Supplementary Figure 14. Treatment- and response-associated tumor immune features.

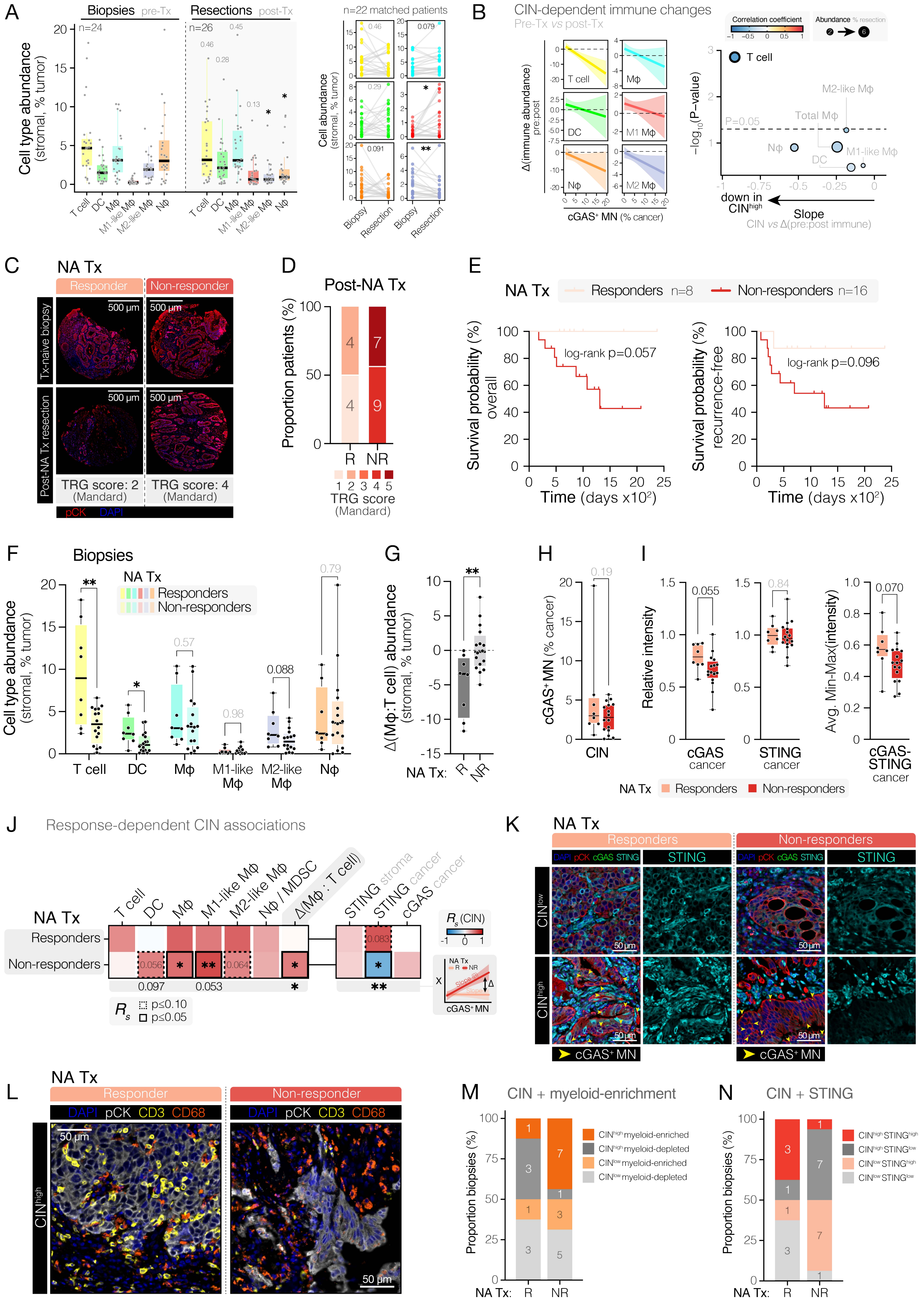

Supplementary Figure 14. Treatment- and response-associated tumor immune features.

(**A**) *Left panel:* Immune cell abundances (immune cell detections in the stromal compartment as a % of all cell detections in the tumor) for all queried immune cell types in pre-neoadjuvant treated (Tx) biopsies and post-Tx resections. Boxes: median ± IQR. Whiskers: Tukey's. Mann-Whitney U test.
Right panel: Immune cell abundances of matched pre-Tx (biopsies) and post-Tx (resections) EAC samples. Wilcoxon matched-pairs signed-rank test.
(**B**) *Left panel:* Scatter plots showing the relationship between baseline CIN (cGAS$^+$ MN frequency in pre-Tx biopsies) and the difference in intratumoral immune cell abundance between pre- and post-treatment samples (Δpre:post) for indicated immune cell types. Regression line and 95% CI shown.
Right panel: Volcano plot summarizing correlation statistics of regressions shown in the left panel. Y-axis: $-\log_{10}$-transformed Pearson correlation p-value. X-axis: slope of the association between CIN and the pre:post difference in immune cell abundance. Dot size: mean abundance of a given immune cell type across post-Tx resections. Dot color: Pearson coefficient.

(**C**) Representative high-resolution images of matched EAC biopsy and resection specimens from neo-adjuvant treatment (NA Tx) responders and non-responders. Stained for DAPI (DNA) and pan-cytokeratin (pCK). Tumor regression grade (TRG) indicated for each patient. Scale bar, 500 μM.

(**D**) Bar plots showing the number of patients classed as NA Tx histopathological responders (R) or non-responders (NR) and proportion of tumors classed as TRG 1–5.

(**E**) Kaplan–Meier plots of overall survival (left panel) and recurrence-free survival (right panel) of NA Tx responders and non-responders. The significance determined using the log-rank test.

(**F**) Immune cell abundances (immune cell detections in the stromal compartment as a % of all cell detections in the tumor) for all queried immune cell types in biopsies of NA Tx responders versus non-responders.

(**G**) Relative difference between intratumoral macrophages (Mφ; CD68$^+$) and T cells (CD3$^+$; Δ[Mφ: T cell]) in biopsy tumors of NA Tx responders versus non-responders.

(**H**, **I**) Box plots of (**H**) tumoral cGAS$^+$ MN frequencies and (**I**) cGAS–STING pathway component expression in biopsy tumors of NA Tx responders versus non-responders.

(**F–I**) Boxes: median ± IQR. Whiskers: minimum–maximum. Unpaired two-tailed t-test.

(**J**) Heatmap of correlations between cGAS$^+$ MN frequency and immune cell abundance or cGAS–STING component expression. Significance of difference between slopes in NA Tx responder versus non-responder patients tested using a linear interaction model; reported at the bottom. Color: Spearman coefficient (R_s).

(**K**) Representative high-resolution images of CIN^{high} and CIN^{low} human EAC tumor biopsies for NA Tx responders and non-responders, stained with DAPI (DNA), as well as anti-pan-cytokeratin (pCK), anti-cGAS and anti-STING antibodies. Scale bars, 50 μM.

(**L**) Example of a CIN^{high} myeloid-dominated NA Tx non-responder tumor and a CIN^{high} myeloid- and T cell-enriched responder tumor.

(**M**) Distribution of EAC tumor biopsies quartilized by CIN and degree of myeloid-enrichment (extent of macrophage:T cell skew) across NA Tx responders and non-responders.

(**N**) Distribution of EAC tumor biopsies quartilized by tumoral STING level and degree of myeloid-enrichment across NA Tx responders and non-responders. **** p ≤0.0001, *** p ≤0.001, ** p ≤0.01, * p ≤0.05.

Supplementary Tables

Supplementary Table 1. Gene signatures.

Supplementary Table 2. TMA patient clinical characteristics.

Supplementary Table 3. IMC antibodies.

Supplementary Table 4. sgRNA sequences.

Supplementary Table 5. Target site sequencing primers.

Supplementary Table 6. Immunoblotting antibodies.

Supplementary Table 7. RT-qPCR primers.

Supplementary Table 8. GSEA pathway groups.

Supplementary Table 9. Multiplex IF antibodies.
